# Joint assembly and genetic mapping of the Atlantic horseshoe crab genome reveals ancient whole genome duplication

**DOI:** 10.1186/2047-217X-3-9

**Published:** 2014-05-14

**Authors:** Carlos W Nossa, Paul Havlak, Jia-Xing Yue, Jie Lv, Kimberly Y Vincent, H Jane Brockmann, Nicholas H Putnam

**Affiliations:** 1 Department of Ecology and Evolutionary Biology, Rice University, P.O. Box 1892, Houston, TX 77251-1892, USA; 2 Department of Biochemistry and Cell Biology, Rice University, P.O. Box 1892, Houston, TX 77251-1892, USA; 3 Department of Biology, University of Florida, P.O. Box 11-8525 Gainesville, FL 32611-8525, USA; 4 Current address: Gene by Gene, Ltd, Houston, TX 77008, USA

**Keywords:** Genotyping-by-sequencing (GBS), Genetic linkage mapping, Genome evolution, Limulus polyphemus

## Abstract

**Background:**

Horseshoe crabs are marine arthropods with a fossil record extending back approximately 450 million years. They exhibit remarkable morphological stability over their long evolutionary history, retaining a number of ancestral arthropod traits, and are often cited as examples of “living fossils.” As arthropods, they belong to the *Ecdysozoa*, an ancient super-phylum whose sequenced genomes (including insects and nematodes) have thus far shown more divergence from the ancestral pattern of eumetazoan genome organization than cnidarians, deuterostomes and lophotrochozoans. However, much of ecdysozoan diversity remains unrepresented in comparative genomic analyses.

**Results:**

Here we apply a new strategy of combined *de novo* assembly and genetic mapping to examine the chromosome-scale genome organization of the Atlantic horseshoe crab, *Limulus polyphemus*. We constructed a genetic linkage map of this 2.7 Gbp genome by sequencing the nuclear DNA of 34 wild-collected, full-sibling embryos and their parents at a mean redundancy of 1.1x per sample. The map includes 84,307 sequence markers grouped into 1,876 distinct genetic intervals and 5,775 candidate conserved protein coding genes.

**Conclusions:**

Comparison with other metazoan genomes shows that the *L. polyphemus* genome preserves ancestral bilaterian linkage groups, and that a common ancestor of modern horseshoe crabs underwent one or more ancient whole genome duplications 300 million years ago, followed by extensive chromosome fusion. These results provide a counter-example to the often noted correlation between whole genome duplication and evolutionary radiations. The new, low-cost genetic mapping method for obtaining a chromosome-scale view of non-model organism genomes that we demonstrate here does not require laboratory culture, and is potentially applicable to a broad range of other species.

## Background

Comparative analysis of genome sequences from diverse metazoans has revealed much about their evolution over hundreds of millions of years. The discovery of extensive gene homology across large evolutionary distances has allowed researchers to track chromosome rearrangements and whole genome duplications. The resulting value of whole chromosome sequences presents a challenge for existing whole genome shotgun (WGS) assembly strategies.

Whole genome duplication events were long suspected [1], but only the availability of genome sequences has allowed confirmation of them in fungal, vertebrate, plant and ciliate lineages [2-5]. In contrast, when only a few chordate, insect and nematode genomes were available, conservation of gene linkage (i.e., synteny) and gene order were observed only between closely-related species, and consequently were not expected to be conserved between phyla. As more metazoan genomes have been sequenced, it has become clear that long-range linkage has been conserved over long time scales in many lineages.

Sequencing the genomes of representatives of chordate, mollusk, annelid, cnidarian, placozoan and sponge clades, has identified 17 or 18 ancestral linkage groups (ALGs) [6-10]. Each of these ALGs consists of a set of ancestral genes whose descendants share conserved synteny in multiple sequenced genomes. These ALGs have been interpreted to correspond to ancestral metazoan chromosomes, and correlations between inferred rates of gene movement between ALGs across the metazoan tree suggest that these ancestral linkage relationships are conserved through the action of selective constraints on a subset of genes [11].

The relatively small number of genomes from anciently distinct metazoan lineages and the fragmented nature of draft genome assemblies still limit both the search for ancient whole genome duplications and the power of the data to constrain models of chromosome-scale genome structure evolution. While WGS sequencing technology and assembly methods are active areas of research and technological development, and have improved at a dramatic pace in recent years, high quality *de novo* assembly of large, complex metazoan genomes remains a difficult and resource-intensive problem. Without genetic or physical maps, or reliance on a high-quality reference genome of a closely-related species, WGS sequencing projects still typically produce assemblies containing thousands of scaffolds, hundreds of scaffolds incorrectly joining sequence from different chromosomes, or both [12].

Next-generation sequencing has greatly reduced the cost of constructing high density genetic maps by eliminating the need to develop and genotype polymorphic markers individually [13]. This has been achieved either by focusing sequence coverage within or adjacent to genomic regions of distinct biochemical character, such as restriction sites with restriction site associated DNA sequencing (RAD-seq) and related methods [14,15], or by combining information across regions using a reference genome sequence [16,17]. While RAD-seq is applicable to organisms lacking a reference genome assembly, it is not directly applicable to comparisons of genome organization across long evolutionary time spans because such comparisons rely on the identification of homologous sequence markers (typically protein-coding genes), which typically have only a small overlap with the restriction-associated markers.

Here we present a genotype-by-sequencing method for constructing a high-density genetic map using low-coverage, low-cost, whole genome sequencing data from the offspring of a wild cross. In this joint assembly and mapping (JAM) approach, the traditionally independent and sequential steps of genome assembly, polymorphic marker identification and genetic map construction are combined. Existing assemblers expect lower densities of sequence polymorphism, deeper coverage, greater computer memory or more aggressive quality trimming that decrease sequence coverage [18-20]. Our current implementation focuses on conservative assembly of short scaffolds sufficient for map construction, but our results suggest that further integration of genetic mapping information within whole genome shotgun assembly methods can be a cost effective way to produce assemblies of large, complex genomes with chromosome-scale contiguity.

We have applied this approach to produce a genetic map of the genome of the Atlantic horseshoe crab, *Limulus polyphemus*. Horseshoe crabs are marine arthropods with a fossil record extending back 450 million years [21]. They exhibit remarkable morphological stability over their long evolutionary history, retaining a number of ancestral arthropod traits [22], and are often cited as examples of “living fossils”. *L. polyphemus* has a genome about 90% the size of the human genome. It is an important species from ecological, commercial and conservation perspectives [23], that has been used as a model system for research in behavioral ecology, physiology and development [24]. The map and SNP markers described here will be a resource for the *L. polyphemus* genome project, research in horseshoe crab population biology, and comparisons of metazoan genome organization. By anchoring protein coding genes to this map, we are able to extend analysis of ancestral linkage groups and whole genome duplications to the chelicerate lineage.

## Data description

A pair of naturally spawning horseshoe crabs and their eggs were collected from their natural habitat on the beach at Seahorse Key. The larvae were hatched at the lab 4 weeks later from the collecting date. The tissue samples from the third walking legs of the parental horseshoe crabs and 34 larvae were used for DNA extraction, library preparation following manufactures’ standard protocols. Two parents and 34 larvae were individually barcoded during library preparation. Illumina paired-end libraries with insert sizes of approximate 300 bp were prepared for each sample. These libraries were pooled together for the subsequent sequncing on the Illumina HiSeq2000 platform at Medical College of Wisconsin Sequencing Service Core Facility. A total of 1.7 billion 100 bp paried-end (PE) reads were obtained after the quality filtering. The total sequencing coverage was estimated as 38.9 × based on the *k*-mer frequency distribution. The raw sequencing data can be retrived from NCBI SRA via NCBI BioProject accession PRJNA187356.

## Analyses

### Assembly and mapping

The JAM method is designed to produce a combined assembly of polymorphic sequences, tagged by genomic regions with a maximum of one single nucleotide polymorphisms (SNP) per *k*-mer window (Methods). Starting with genomic reads from a mating pair of adult *L. polyphemus* and 34 offspring (100 bp paired-end reads on 300 bp inserts), we analyzed 1.7 billion reads containing at least one high-quality 23-mer.

**Figure 1:**
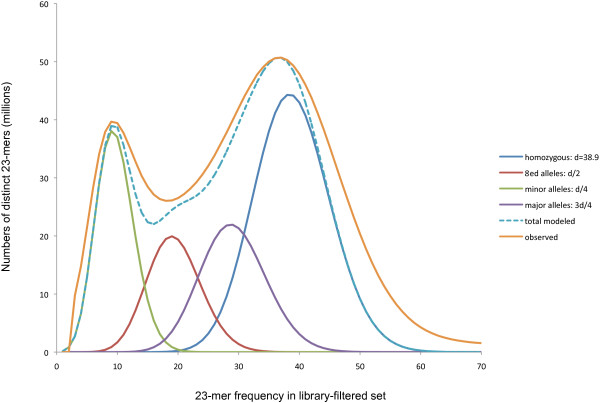
**Fitting Poisson distributions to Limulus 23mer frequencies.** The distribution of sequenced 23-mers, modeled as sampling genomic loci that are homozygous (single allele shared by all haplotypes), have two alleles that are tied (A and a present in parents as AA×aa or Aa×Aa), or have two alleles in a major-minor relationship (present in parents as AA×Aa or Aa×aa). Alleles whose parental contributions are homozygous, tied, major or minor each have a frequency peak corresponding to their distinctive fraction of the overall depth of genomic sequencing d. Loci sharing simple or repetitive sequence not sufficiently unique at a 23-mer scale contribute to a long tail off the right edge of the plot.

Fitting Poisson models for unduplicated sequence to the frequencies of filtered 23-mers suggests that 1.1 billion genomic loci are unique at this resolution (Figure 1). This fit assumes that the vast majority of mappable genomic loci have only one or two alleles represented in the parents’ four haploid contributions, which gives rise to the four components plotted in Figure 1. Of these sufficiently unique loci, 63% are modeled as homozygous, 27% as paired major-minor alleles, and the remaining 10% as tied allele pairs. The corresponding SNPs, if always at least 23 bases apart, would be 1.6% of bases in the unique loci. Dividing the total number of filtered 23-mers by the modeled homozygous depth of coverage *d* = 38.9 yields an estimated genome size of 2.74 billion bases, consistent with the measured DNA content of 2.8 pg (978 Mb ≃ 1 pg DNA) [25].

We categorize specific 23-mers by their edit distances to others: having no neighbors within a single base substitution (unique tags) or with a single mutually unique one-substitution neighbor (“SNPmer pair” tags). A subset of these, including SNPmer pairs for approximately 7.9 million SNPs, constitute the tags used for contigging and scaffolding. The SNP-mer pairs account for approximately 45% of the modeled fraction of alleles, the others missed from similarity to other sequences (e.g., due to repeats) or distance from each other (because of indels or multiple SNPs per 23-mer).

Chaining these 23-mers together (see Methods) produces an initial 6.6 million contigs, 3.9 million of which are linkable by paired reads for scaffolding. Applying Bambus [26] produces 944,000 scaffolds spanning 1.3 billion bases (Table 1). These scaffolds serve as markers incorporating multiple 23-mer tags, including SNPmer pairs used to identify haplotypes.

**Table 1:** ***K*-mer contig and scaffold statistics**

**Assembled**	**Count**	**Total (bp)**	**Avg. span (bp)**	**n50 span (bp)**
*k*-mer contigs	6,614,434	1,240,275,515	188	418
Linkable contigs	3,925,844	1,137,576,911	290	460
Initial scaffolds	944,246	1,261,263,172	1336	3047
Reference scaffolds	944,246	1,295,334,515	1372	2930
Reference bases		1,131,458,744	1198	2553

**Figure 2:**
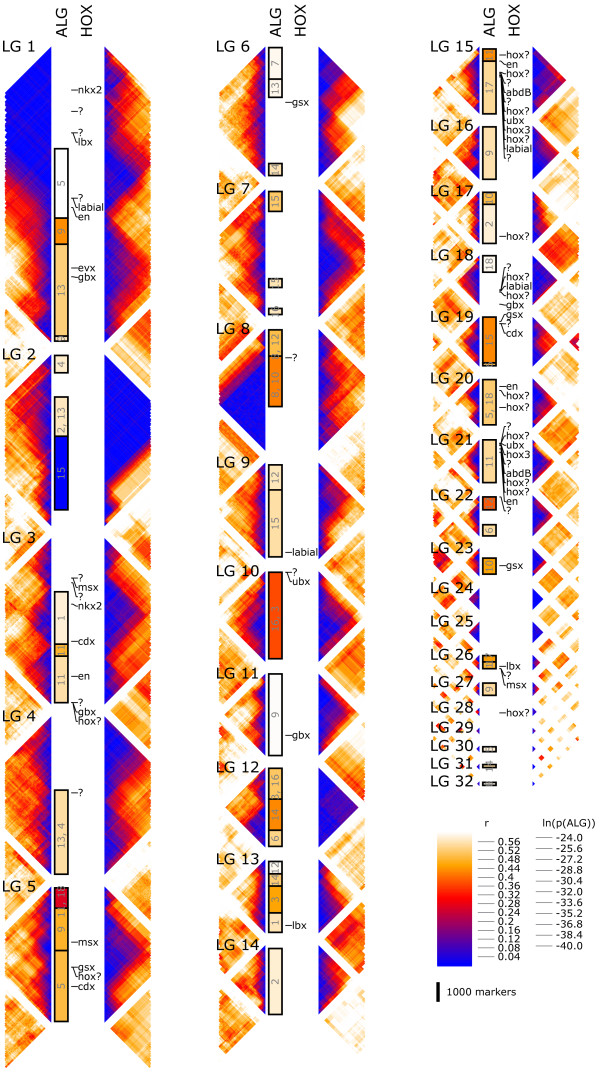
**Each of the numbered blocks represents one of the 32 linkage groups of the *L. polyphemus *genetic map, and is composed of four columns: Two bands of the triangular matrices in which the color scale indicates the fraction of samples showing recombination between pairs of markers; maternal recombination frequency is shown on the left, paternal on the right.** A column labeled “ALG” indicates segments of significant (*p*  < 0.05 in Fisher’s Exact Test, after Bonferroni correction for multiple tests) conservation of gene content with ancestral bilaterian linage groups. The column labeled HOX shows the map positions and types of predicted homeobox transcription factor genes. The two color scales are for: recombination frequency between pairs of markers and log p-value for enrichment in gene content with ancestral linkage groups.

After assembly, the mean density of SNPs across the four parental haplotypes in assembled regions was estimated based on read re-alignments to be 7.6 per thousand bases. We jointly inferred the phases of these SNPs and segregation pattern (offspring genotypes) in the mapping cross for each marker in a maximum likelihood framework (Methods). We focused on the 91,320 markers with at least 18 inferred bi-allelic SNPs for constructing the linkage map. These markers grouped into 1,908 high-confidence map bins (i.e., unique segregation patterns, assumed to correspond to loci in the genome uninterrupted by meiotic recombination in the cross [27]. Map bins fell into 32 linkage groups (Figure 2), close to the 26 pairs (2 N = 52) previously found in a cytogenetic analysis of two chromosome spreads [28]. Twenty map bins were removed for having inconsistent positions in the maternal and paternal maps, and 12 were singletons.

To estimate the frequency of incorrect genotype calls as a function of the log likelihood difference between the called and alternative genotype (genotype confidence score), including contributions from uncertainty in SNP-mer identification, assembly and sampling noise, we carried out a simulation of the library pooling and sequencing, *k*-mer assembly and genotype inference protocols, using the sequenced *Ciona intestinalis* genome as a starting point.

**Figure 3:**
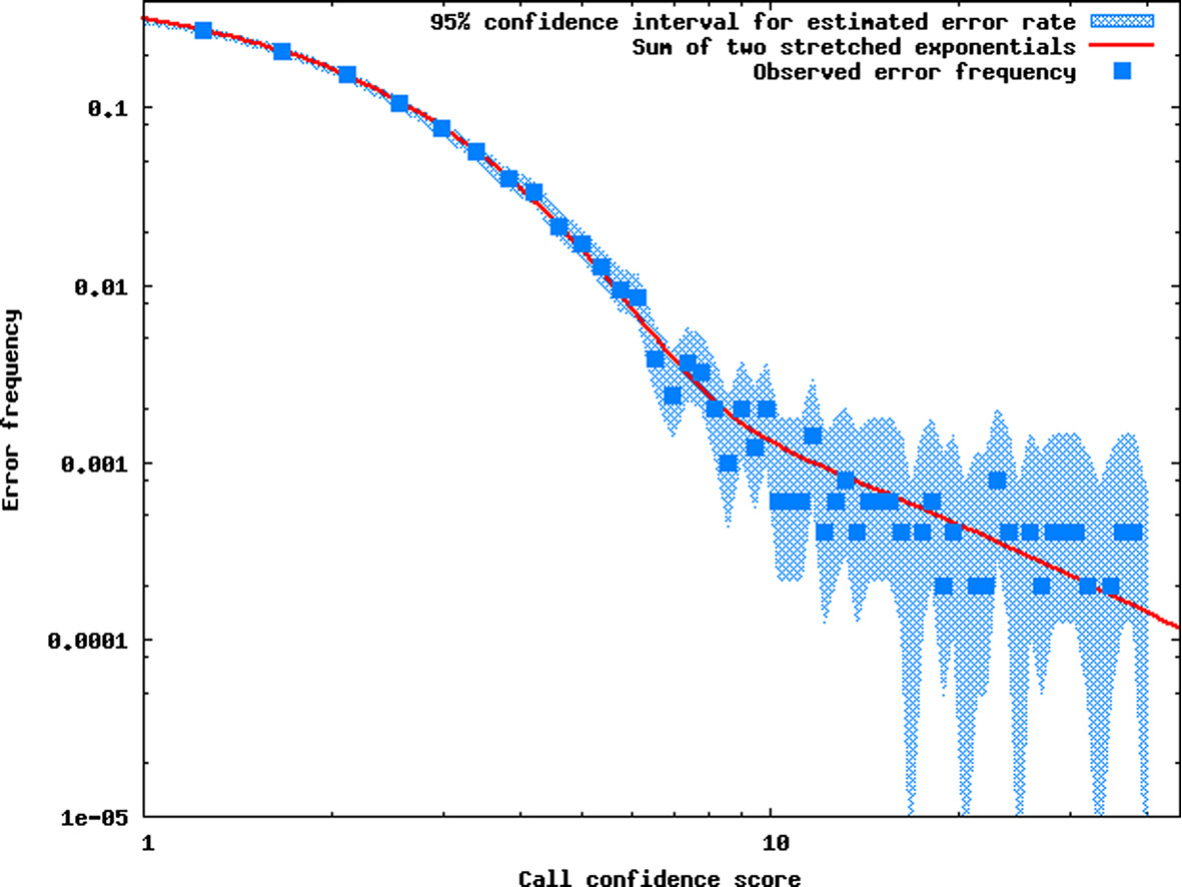
**Genotype call error rate as a function of call confidence score for bins of 10,000 calls in simulated *Ciona intestinalis *genome data.** The stippled blue region shows 95% confidence intervals of the Bayesian posterior probability distribution of the underlying error rate computed from the Beta distribution *Beta*(*n*_*e*_ + 1, *n*_*c*_ − *n*_*e*_ + 1) conjugate the assumed binomial distribution of observed errors, where *n*_*e*_ and *n*_*c*_ are the number of errors and number of calls in each bin respectively. The red curve shows the best fit error model $a_{1\;}e^{-\frac{s^{c_{1}}}{b_{1}}}+a_{2}e^{-\frac{s^{c_{2}}}{b_{2}}}$, with parameter values *a*_1_ = 0.49, *b*_1_ = 2.08, *c*_1_ = 1.26, *a*_2_ = 5.47, *b*_2_ = 0.17, *c*_2_ = 0.16. *χ*^2^ reduced = 0.82.

In the simulated *C. intestinalis* data set (Methods), a single stretched exponential distribution provided a good fit to the frequency of genotype calling errors as a function of the call confidence score for scores up to 6, or down to error frequency of approximately 1%. The observed error frequency declined more slowly for higher confidence scores. The minimum *x*^2^ fit used for estimating the genotyping error rate in the *L. polyphemus* map bins was $p_{e}\left(s\right)=a_{1\;}e^{-\frac{s^{c_{1}}}{b_{1}}}+a_{2}e^{-\frac{s^{c_{2}}}{b_{2}}}$, with parameter values *a*_1_ = 0.49, *b*_1_ = 2.08, *c*_1_ = 1.26, *a*_2_ = 5.47, *b*_2_ = 0.17, *c*_2_ = 0.16 (Figure 3).

Applying this model to the *L. polyphemus* marker genome calls, we estimated that the genotype calling error rate in the map bin representative markers was 0.0099. We observed that 51% of adjacent map bin pairs are separated by a single inferred recombination event in the cross, and 94% are separated by three or fewer recombinants in each parent.

Of the 91,320 markers with at least 18 putative SNPs, 84,307 (92%) were assigned to their closest map bins with a threshold of (Methods), for an estimated genome-wide average density of one mapped sequence marker every 32 kb. A mean of 45 markers were mapped to each map bin, and the number of markers mapped was used to estimate the relative physical size of map bins. Approximately 46% of the scaffolds with 12–17 SNPs could be placed with the same threshold, for an additional 32,688 markers, or one marker every 23 kb.

The total length of the scaffolds assigned to map bins was 411 Mb, and they contained 2.67 million bi-allelic SNPs assigned a phase with a posterior probability of at least 0.99. Of these, 72% were inferred to be unique to one of the four parental chromosomes. This is close to the 74% predicted under the finite sites neutral coalescent model given the observed SNP density [29].

### Sequence composition and recombination rate

In the scaffolds longer than 1 kb (N = 378,506 and mean length = 2.9 kb), the *G*/*C* base content was 33.3 ± 2.8%, and the local relative frequency of CpG dinucleotides was bimodally distributed, with about 30% of sequences exhibiting depletion of CpG. TpG and CpA dinucleotides were over-represented on average and their local densities negatively correlated (r = −0.54, p  < 2.2e-16) with CpG density, suggesting ongoing germ-line CpG methylation for a fraction of the genome [30].

The mean maternal and paternal recombination rates were estimated to be 1.28 and 0.76 centimorgans per megabase respectively, consistent with expectation based on the negative correlation between recombination intensity and genome size observed in previous studies [31]. We did not observe evidence of segregation distortion for any map bins. The correlation in local recombination rates in two parents across the genome is estimated as *r*^2^ = 7.1%; *p*  < 1e-29, suggesting considerable variation in recombination landscape between two sexes in limulus [32-34]. A positive correlation between local recombination rate and local SNP density was observed (*r*^2^ = 9.7% ; *p*  < 1e-40), which is consistent with previous observations in human with comparable correlation coefficient [35].

### Ancestral linkage group conservation

**Figure 4:**
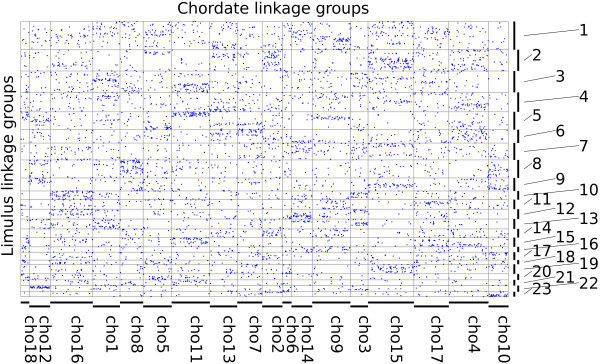
**Limulus-Human macro-synteny dot plot.** Blue points indicate the position of human genes in reconstructed ancestral chordate ALGs (vertical displacement) and their candidate orthologs in the 30 *L. polyphemus* linkage groups (horizontal displacement).

**Figure 5:**
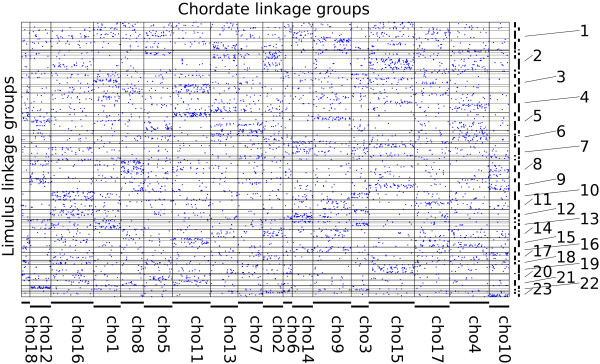
**Limulus-Human macro-synteny dot plot as in Figure**4**, showing breaks introduced by hidden Markov model segmentation of the linkage groups as vertical lines.**

We found that 34,942 scaffolds have significant sequence conservation with 10,399 predicted proteins of the tick *Ixodes scapularis*: like *Limulus*, a chelicerate, but one with a well-annotated genome [36]. 6,246 of these hits formed reciprocal best pairs, of which 5,775 (92%) could be placed on the linkage map at a threshold of *p* <. These were used as conserved markers for comparisons of genome organization. When linkage groups were divided into 108 non-overlapping bins of 1,000 markers, 52 had significant (*p*  < 0.05, after Bonferroni correction for 1,944 pairwise tests) enrichment in shared orthologs (or “hit”) with at least one of eighteen ancestral chordate linkage groups [7]. A hidden Markov model segmentation algorithm [6] identified 40 breakpoints in ALG composition in the linkage groups. Approximately 72% of the genome is spanned by 53 intervening segments that hit one or (for eight of them) two ALGs (Figures 4 and 5). Each of the eighteen ancestral ALGs has at least one hit among the 45 segments with a unique hit to the ALGs.

### Whole genome duplications

Whole genome duplication (WGD), or polyploidization is a rare, but dramatic genetic mutation event which doubles the size of a genome and creates a redundant pair of copies from every gene. Because it creates redundant copies of genes for entire biochemical pathways and genetic networks, it has been proposed that it creates unique raw material for the evolution of novel biological functions and increased complexity.

### Homeobox gene clusters

Homeobox genes encode a large family of transcription factors involved in diverse embryonic patterning and structure formation processes of eukaryotes. As a particular subfamily of homeobox genes, the Hox cluster is known to control metazoan body patterning along the anterior-posterior axis. We identified 155 scaffolds with significant homology to predicted chelicerate homeobox gene sequences in public databases. We classified these sequences into homeobox subfamilies (Methods) and placed them on the map by best hit. Two large clusters of Hox genes are found on linkage group (LG) 15 and LG 21, each containing multiple members of the anterior, central and posterior classes. There are also two parahox cluster homologs, each with three homeobox genes: *gsx* and *cdx* orthologs and a third homeobox gene not confidently assigned to a subfamily in our analysis (LG 5 and LG 19). There are two smaller clusters containing multiple hox genes (LG 18 and LG 20), and clusters of other homeobox genes, including members of the *msx*, *lbx*, *nk*, *evx* and *gbx* families (Figure 2).

### Genomic distribution of paralogous genes

**Figure 6:**
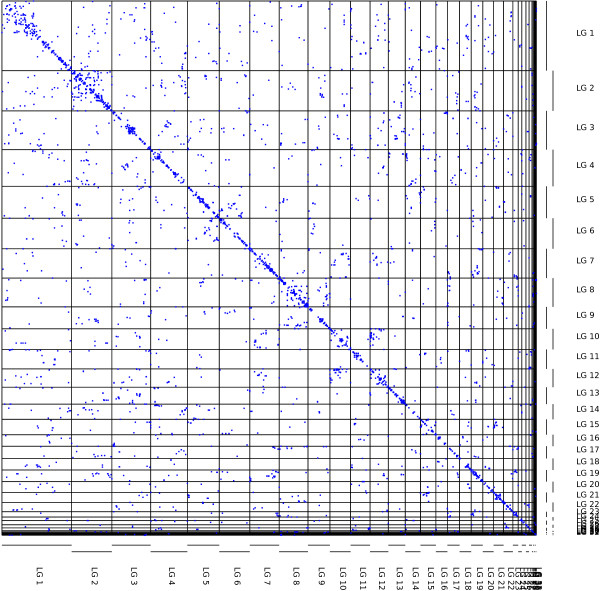
**Genomic distribution of candidate paralogs.** The map positions of pairs of putatively paralogous protein coding genes within the *L. polyphemus* genome are plotted with blue points. Pairs are biased toward nearby map positions, and therefore concentrated along the diagonal. Also, paralogs split between linkage groups are significantly clustered into “paralogons”.

WGD creates many pairs of duplicate genes or “paralogs”. The distinctive features of these genes have been used to infer WGD events in fungal, vertebrate and plant genomes [2-4]. We examined the genomic distribution of 2,716 pairs of candidate paralogous gene markers in *L. polyphemus* for signatures of WGD. In 45% of these pairs both markers mapped to the same chromosome, compared with 5.3 ± 0.5% in 1,000 datasets with randomly-permuted paralogous gene identities. The mapped positions of pairs within the chromosomes were highly correlated (average *r*^2^ = 0.81, and exceeding 0.95 for 8 of the large chromosomes; Figure 6), suggesting that many of the pairs represent recent tandem gene duplicates or single genes fragmented across multiple markers. In the following, these same-chromosome paralogs are referred to as “tandem” duplicates.

Inter-chromosomal duplicates are clustered into conserved paralogous micro-synteny blocks (or “paralogons” [3]): there are 25 pairs of loci, each with at least six (*mp* = 6) independent paralog pairs clustered with a maximum gap (max-gap) of 300 markers between adjacent paralogs in each cluster. Paralog pairs are considered independent if they are based on homology to a distinct out-group gene, to guard against relying on either multiple exons of the same gene, or recently-duplicated genes as independent evidence of ancient segmental paralogy. These clusters span 25,044 markers, or 30% of the map, after removing redundancy from paralogons with overlapping footprints. In 1,000 datasets with randomly-permuted paralogous gene identities, the maximum number of such clusters observed was 11; the mean and standard deviation were 3.9 ± 1.0. The observed clustering into paralogons was greater than that in the randomized datasets over a broad range of choices of max-gap and m_p_. For example, for max-gap = 100, *mp* = 3 there are 52 clusters vs. 3.5 ± 1.9, range 0–10; for max-gap = 500, *mp* = 9 there are 12 vs. 2.9 ± 1.7, range 0–9. Because of the large proportion of apparent tandem gene duplicates (45%), this randomization scheme increases the number of inter-chromosomal paralog pairs relative to the data, making it a conservative significance test for inter-chromosomal paralog clustering. When genes with tandem duplicates are excluded from the randomizations, the observed number of clusters is greater than the maximum observed in 1000 randomizations for all the combinations of max-gap in the set (100, 200, 300, 400, 500, 600) and *mp* in the set (3, 4, 5, 6, 7, 8, 9). 23 max-gap = 600, *mp* = 7 clusters span 59% of the map, compared to respective mean number and map coverage of 3.3 ± 1.8, and 13 ± 6% in these randomizations.

Among the marker pairs mapping to different chromosomes, we found a significant excess of pairs relating segments derived from the same ALG relative to randomization controls (247 pairs vs. 102 ± 11, *p*  < 0.001 in randomizations of all genes; 202 vs. 46 ± 7 when genes in tandem duplicates are excluded). This pattern is consistent with the creation of these segments by duplication (rather than fission).

The max-gap clusters have a significant amount of overlap among their footprints. For example, the footprints of the max-gap = 600, *mp* = 7 clusters had a total length of 72,072 markers, but a net footprint after redundnacy removal of 49,545 markers. A genomic region *a* which has conserved synteny with two other regions of the genome (*b* and *c*) could arise either from mixing of adjacent regions (through local genome rearragements) with homology to *b* and *c* respectively, or by successive duplications. In the latter case *b* and *c* are also homologous. We examined the relationships among the paralogons for evidence of successive rounds of duplication. We considered a graph in which nodes correspond to merged, non-redundant paralogon footprint regions. Nodes are connected with edges if a max-gap cluster connects the two nodes. The average clustering coefficient of this graph is equal to the probability that footprints *a* and *c* share a max-gap cluster, given that there are edges (*a*, *b*) and (*b*, *c*) in the graph. We compared the clustering coefficients to those found in random Erdős-Rényi graphs with the same number of nodes and edge probability as the observed graph. We found that the observed data shows significantly more clustering than these random graphs for a wide range of choices of max-gap and *m*_*p*_. For example, for max-gap 600, *m*_*p*_ =7, the average clustering coefficient is 0.19, while 10,000 random graphs had coefficients of 0.034 ± 0.042, *p* = 0.0039.

### Age distribution of paralogous genes

**Figure 7:**
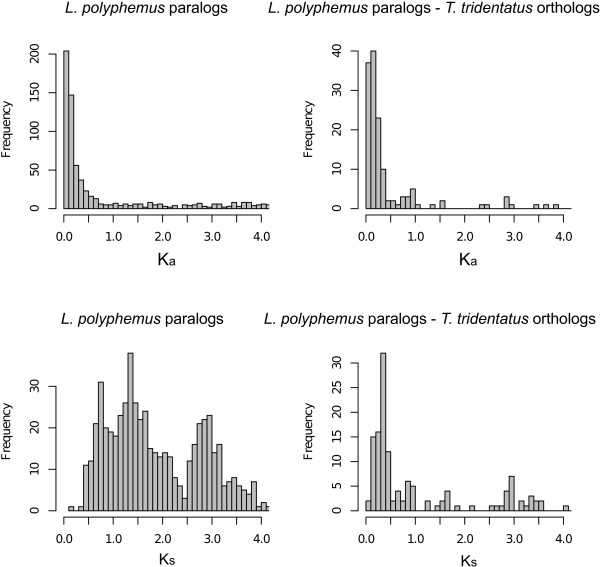
**Distribution of estimated non-synonymous (K**_**a**_**; top) and synonymous (K**_**s**_**; bottom) and sequence divergence rates for pairs of putative *L. polyphemus *** **paralogs (left) and *L. polyphemus *- *T. tridentatus *** **orthologs (right).**

Because WGD events create many paralogs at the same time, they leave characteristic peaks in the age distribution of paralogous genes. In *L. polyphemus* the distribution shows peaks centered at 0.71 and 1.34 substitutions per synonymous site (K_s_), values within the approximately linear response range of K_s_ estimates to WGD age [37] (Figure 7). For comparison, the synonymous site divergence between an Asian horseshoe crab species *Tachypleus tridentatus* and *L. polyphemus* has a mode of 0.35. The common ancestor of these species has been estimated to have lived 114–154 million years ago (MYA), coincident with the opening of the Atlantic ocean [38], suggesting a WGD event 230–310 MYA, and possibly an older one 450–600 MYA.

## Discussion

Our results demonstrate that a low cost, combined approach to whole genome sequencing and genetic mapping can be used to efficiently create a very high density genetic recombination map for a non-model organism with a large genome. Because the approach uses genome-wide sequencing, a large number of sequence markers can be anchored to the map, allowing comparisons of genome organization at the chromosome scale over very large evolutionary divergences. The identification of chromosomal segments with significant gene composition homology to each of the chordate ALGs demonstrates that the predominance of fusion and mixing of ancestral linkage groups previously observed in analyzed ecdysozoan genomes [10] is not ancestral to, or universal in the clade.

The map allows quantitative characterization of other features of chromosome-scale organization, such as the correlation between local recombination rate and polymorphism levels. Similar positive correlations between local recombination rate and polymorphism level have been observed in other metazoans including humans [39-41] and plants [42,43]. Future comparisons with more closely related chelicerates will allow tests to distinguish whether these rates are positively correlated with inter-specific divergence, consistent with a neutral process of correlated mutation and recombination rates [35]. Alternatively, the association could be explained by hitchhiking and background selection [44].

The enrichment of inter-chromosomal paralog pairs in segments of the same ALG origin is consistent with their creation by duplication (rather than fission), although because small-scale duplication is biased toward local (tandem) duplication, fission of segments could also leave behind an enrichment of paralogs. Such a mechanism, however, would not create the observed organization of paralogs, that is, their clustering into “paralogons”. The fact that these paralogons span a large portion of the map (59%) suggests that it was a whole genome duplication, rather than segmental duplications that gave rise to the pattern.

The existence of duplicated hox and parahox clusters on four different chromosomes is highly suggestive of multiple whole genome duplication. Hox clusters have not been found in duplicate copies except in vertebrates where they have been created by whole genome duplication, and have only rarely been subsequently lost.

The double-peaked shape of the distribution of synonymous site divergence between pairs of paralogs, combined with the existence of two small clusters of HOX genes in addition to the two complete HOX clusters suggests that there may have been two rounds of whole genome duplication in the horseshoe crab lineage.

WGDs preceded major species radiations in vertebrates, angiosperms and teleost fish, and the importance of their role in evolution is the subject of long-running debate [1-4]. The discovery of whole genome duplication in an invertebrate, and during horseshoe crabs’ long and famously conservative evolutionary history suggests that such events may have been more common than previously assumed in metazoan evolution, and that while they may have provided raw material for adaptive evolution in some cases, they are not evolutionary drivers.

## Methods

### Joint assembly and mapping (JAM) overview

Barcoded genomic DNA libraries were created, pooled, and sequenced in four lanes on the Illumina HiSeq2000 platform for a mating pair of *L. polyphemus* and 34 offspring.

The JAM method proceeds through three major phases: 1. The frequencies of DNA sub-sequences of fixed length *k* (*k*-mers) are profiled to characterize the quality, uniqueness, polymorphisms and repetition in genomic reads, using software we developed building on work from the Atlas assembler [45]. Allelic pairs of *k*-mers representing alternate forms of SNPs are identified and tracked through the subsequent steps. 2. Contigs are assembled on a graph of unique *k*-mers and paired SNP *k*-mers sampled to reduce memory usage, then ordered and oriented using the Bambus scaffolder [26,46]. Each multi-SNP scaffold is treated as a single marker for the linkage mapping steps 3. The paired SNP *k*-mers (in each scaffold are combined with the read, mate-pair, and parent- or offspring-library associations of their alleles for haplotype phasing and construction of a high density genetic linkage map. The software is public available as open source software at GitHub [47].

### Sampling and sequencing

The parental horseshoe crabs and their eggs were collected from their natural habitat on the beach at Seahorse Key, an island along the west coast of north Florida, on 27 March 2010. This naturally spawning pair were observed as the eggs were being laid and fertilized (fertilization is external in this species, i.e., the eggs are fertilized in the sand under the female as the eggs are being laid). The tissue sample were collected from the third walking legs of this parental pair. We marked where the egg samples were laid and returned a few hours later and dug up the nest, then removed the fertilized eggs. We also have conducted paternity analyses that show that fertilization is by the associated male and not by extraneous sperm that might be at the nesting site (in this case the density of nesting females was low on this day so we know that the eggs we collected were from the pair we observed). Trilobite larvae were reared in plastic dishes as previously described and hatched from the eggs 4 weeks later [48]. Tissue samples and larvae were preserved in RNALater. Genomic DNA purification and library construction were carried out using Qiagen DNAEasy, Illumina TruSeq and Nextera kits, following manufacturers’ protocols. Barcoded samples were pooled and sequenced on the Illumina HiSeq2000 platform.

*Limulus* larvae were processed as follows; each larva, suspended in 100 μ*L* of RNAlater and stored at −80°C in a 1.5 mL Eppendorf tube, was thawed on ice, after which RNAlater was removed. DNA was extracted using the Qiagen DNAEasy kit per manufacturer’s protocols. DNA was quantified using picogreen DNA quantitation kit. To prepare TruSeq libraries, DNA was first purified another time using zymo genomic DNA clean columns per manufacturer’s protocols. Adult *L. polyphemus* DNA was prepared as above, but using claw tissue rather than whole larvae. All DNA extracts were tested by gel electrophoresis to ensure DNA was not degraded. TruSeq libraries were prepared at University of Georgia’s Georgia Genomics Facility. 1–5 μg of sample DNA was subjected to fragmentation using Covaris sonicator. Fragmented DNA was then used for library construction using Illumina TruSeq library prep kits. Libraries were pooled together in equimolar amounts (for 10 larvae) and used for the first sequencing run in separate lanes for the parental and larval pools. For larval samples 11–34, library prep was switched from TruSeq to Nextera kits. Nextera library preparation was performed according to manufacturer’s protocol. The Nextera library product was quantified by picogreen, and fragment size distribution was checked by using Lonza flash gel, to ensure that fragment size distribution was between 300–1,000 bp. Sample libraries were pooled in equimolar concentrations and sent for the second sequencing run in two lanes, each on a pool of 12 larvae. Both sequencing runs, comprising four library pools, were performed on the Illumina HiSeq2000 platform at Medical College of Wisconsin Sequencing Service Core Facility.

A total of 1.7 billion Illumina reads qualified for *k-*mer analysis and assembly by containing at least 23 consecutive q20 bases. The maternal library accounted for 13% of these reads, the paternal library 7.4%, and the 34 offspring libraries for 2.4% on average, 0.64% at minimum.

### k-mer decomposition

We determined a lower bound on the *k*-mer size long enough for a given expectation of uniquenes in a random genome. While increasing *k* reduced the rate of coincidentally repeated *k*-mers, it also reduced the effective depth of coverage due to untrimmed errors and edge effects at read ends — and increased the cases of multiple SNPs per *k*-mer locus, which are not tracked in our current software implementation. We can approximately model a genome-scale string *G* of random nucleotides as *G* samples taken with uniform probability from the space of all *k*-mers (of size 4^*k*^/2 for odd *k*-mers treating reverse-complements as same; slightly more for even *k*). The Poisson distribution then gives the probability that a location in *G* has its own, unique *k*-mer (shared with no other location) as

$$e^{-\lambda },\;\mathrm{where}\;\lambda =\frac{G}{\frac{4^{k}}{2}}=\frac{2G}{4^{k}}$$

The probability of a location sharing its *kmer* is then; thus, to limit the maximum rate *R* of *G*-locations sharing *k*-mers, we require *k* ≥ [log_4_(−2*G*/ln(1 − *R*))]. For example, for a mammalian-scale genome of approximately 3 billion bases, and *R* = 0.1%, we chose *k* ≥ 22. For *Limulus polyphemus*, the Animal Genome Size Database [25] reports an estimated haploid genome size of 2.80 pg and, as each picogram represents almost a billion nucleotide base-pairs of DNA, the mammalian-scale choice of *k* applies [19,49].

This lower bound ignores chemical and biological sequence biases, so selecting *k* for a real genome project requires attention to error rates, repeats tandem and interspersed, and genome size, all known vaguely, if at all, before sequencing. Studying the *k*-mer distributions after sequencing can clarify these genomic properties as we select *k* to maximize the net yield of candidate *k*-mer tags, between errors and with at most one SNP location, in sequencing reads. We converted Illumina/Solexa FASTQ format (paying attention to the different quality encodings of the software versions) into FASTA format [50], masking (replacing with ‘N’) any base with Phred-scale [51] quality below 20, and soft-masking (representing in lower case) other bases with quality below 30. For initial trimming experiments, we varied these quality thresholds as indicated below. We stored *k*-mers in hash tables with open addressing [52], supporting odd *k*  < =31. We tallied for each *k*-mer a bit vector for presence or absence in up to 64 sample libraries (36 for *Limulus* parents and offspring), and an overall count of occurrences in all libraries (count limited to 64 − 2 *k* bits). Where the *k-*mer hash would be too large for available memories, we sampled the *k*-mers using a hash-slicing factor *S* (must be prime). Representing each *k*-mer as an integer in, slice *s* consists of those *k-*mers whose remainder on division by *S* is *s*. We can tabulate one slice for a representative sample of 1/*S k-*mers (for initial estimation of depth of coverage and genome size) or, using *S* independent jobs, to collect information for *k*-mers in all slices. Our hash tables stored odd-length *k*-mers so that reverse-complementary sequences can be combined without the ambiguous orientation of palindromic sequences (e.g., ATCGAT).

After selecting *k* as described above and making a full tabulation of *k*-mer counts and bit vectors, we filtered out *k*-mers not expected to represent genomic sequence. *k*-mers were required to have three copies in the total sequence set, with at least one copy in the initial run and one in the second run. This was partly to filter out incomplete adapter sequences, which can be difficult to trim, but were different in the two runs.

Extending methods developed for the Atlas assembler [45] to heterozygous sequences, Figure 1 gives a rough decomposition of the *k*-mer frequency distribution for 23-mers with quality ≥20, minimizing the square of the residuals of *k*-mer counts on frequencies 3 through 70 while not exceeding the observed counts. Four linked distributions model fractions of the genome as monoallelic or biallelic: homozygous regions with *d* = 38.9-fold coverage (dark blue), minor alleles covered at *d*/4 (green), tied alleles at *d*/2 (red) and major alleles at 3*d*/4 (purple). This fit is robust enough to confirm the abundance of major-minor allele pairs (27% of *k*-loci, vs. 10% for tied alleles), with the broader peaks in the data than in the fitted curves consistent with less uniform sampling (for example, varying coverage of parents and offspring). The Poisson decomposition suggests a density of polymorphisms of 1.2% in major-minor allele pairs, based on dividing the modeled number of such sequenced pairs by *k* (assuming most polymorphisms are SNPs spaced at least *k* bases apart), by *d* (the estimated depth of sequencing) and by the estimated genome size of 2.74 billion bases.

### SNPmer identification

The filtered kmer counts, computed in parallel, are loaded into a hash table with additional fields to track kmers that are uniquely within one mismatch of each other. Because this step analyzes all (non-error) *k*-mers in one table, this requires a single large-memory processor (on the order of 32 GiB).

For each *k*-mer, we check all its 3 *k* one-substitution neighbors. The *k*-mers are partitioned each into one of three categories: *unique*: having no edit-neighbors within one substitution; *ambiguous*: having either multiple one-substitution neighbors, or one neighbor that has multiple neighbors; or *partnered*: uniquely pairable with exactly one other *k*-mer differing by one substitution, such *k*-mers also known from now on as SNPmers or SNPmer pairs. For each SNPmer, we save the position of the substitution, a bitmask for the change (transition, complement, or non-complement transversion), and whether the canonical form of the partner in the table has the same sense or is reverse complemented with respect to this *k*-mer.

Only partnered and unique *k*-mers will be further tracked. While this limited method cannot identify *k*-mers for genomic SNP and non-SNP locations with complete confidence, false pairing or missed pairing should have limited effects, as confirmed by assembly experiments with simulated *Ciona* sequence (see *Error model calibration* in Methods). *False pairing*, due to coincidental similarities or repeats, would combine nodes of the *k*-mer graph (see below) and cause noise in the scaffolding, haplotype phasing, and linkage analysis. Such misleading links are minimized by the *robust edge* requirements in contigging and scaffolding, described below. *Missed pairing* can happen from indel polymorphisms, SNPs separated by fewer than *k* − 1 positions, failure to sequence minor alleles, or ambiguity due to too many similar *k*-mers. Ambiguously non-unique *k*-mers will be skipped over (reducing connectivity of the *k*-mer graph if there are too many in a row). Where allelic *k*-mers misidentified as unique cause conflicting edges in the *k*-mer graph, nodes for unpartnered major alleles will either be chained into contigs with flanking unique sequence or left as orphaned fragments, and unpartnered minor alleles will be left as orphaned fragments. Overall, errors in identifying parnered and unique *k-*mers should shorten contigs and scaffolds and hide linkage, not promote false linkages.

Table 2 shows the totals and percentages of the different kmer categories, counting each SNPmer pair as one *k*-mer. SNPmer pairs account for 16.3% of the putative genomically unique 23mer markers; dividing by 23 gives us the fraction of bases in those markers that are putative SNPs: 0.71% .

**Table 2:** **K-mer categories, counting a SNPmer pair as one *k *-mer**

***k*-mer type**	**#Distinct**	**Percentage**
No partner/unique	946,431,901	55.48%
Partnered/SNPmer	184,756,149	10.83%
Ambiguous	574,557,296	33.68%
TOTAL	1,705,745,346	

### Node k-mer selection

To reduce the memory requirements of our *k*-mer assembly graph, we select a approximate one-tenth subset of the SNPmer and unique *k*-mer tags.

In the case of a true SNP at least *k-*1 bases from other SNPs and from gaps in error-free coverage of either allele, there will be *k* covering SNPmer pairs (provided that covering *k*-mers are also uniquely pairable). By taking only SNPmer pairs with the substitution in particular positions, we can reduce the size of the graph and its redundancy. Analyzing the distribution of substituted position for all the SNPmer pairs, we observe an enrichment for substitutions near the ends, probably due to proximity to low-quality sequence. By selecting positions 3, 12 and 21 of 23-base SNPmers, we avoid the most problematic positions and reduce this portion of *k*-mer nodes by a factor of 7.67.

Unlike for SNPmers, there are no canonical positions that identify the unique, unpaired *k*-mers . Several mechanisms have been proposed for sampling *k*-mers in a representative way [53,54]. We use the more pseudo-random hash-slicing rule, already discussed above, to sample a single slice of *k*-mers: those whose integer encodings are congruent to a particular slice number *s*, modulo *S* (the hash slicing factor). We have found that on the finished human genome (results not shown), hash slicing is effectively a Poisson sampling, with sampled *k*-mers spaced according to an exponential distribution.

A caveat in applying hash slicing is that taking the remainder modulo a prime is not very pseudo-random for Mersenne primes (equal to 2^*p* − 1^ for some *p*), when *k*-mers are represented in base-4 encoding [52]. We therefore pick a slicing factor of 11, the smallest non-Mersenne prime greater than our SNPmer sampling factor.

The resulting *k*-mer subset has 86.0 million unique-unpaired *k*-mers and 24.0 million SNPmer pairs, each reduced as predicted, for a total factor of 9.8 reduction in *k*-mer nodes for the next step.

### Contigging and scaffolding

Each 23mer tag (unique *k*-mer or SNPmer pair) in the above subset is a node in the *k*-mer graph. Nodes are connected when the corresponding *k*-mers appear consecutively in at least one read of the input (any intervening *k*-mers having been skipped due to sampling or ambiguity). The relative orientation, distance and number of supporting reads of the *k*-mers is stored in the edge. When conflicting distance or relative orientation is observed among different reads for the same pair of *k*-mer nodes, all edges from both nodes in the corresponding direction are ignored in contigging.

The nodes of the *k-*mer graph represent DNA tags and have distinct upstream and downstream ends. One edge at each end of a node is identified as *robust* if supported by a supermajority of the reads for all edges in that direction: the number of supporting reads is greater than or equal to both (1) two plus the sum of the read counts for all other edges in that direction and (2) twice the read count of the next-most supported edge in the same direction. By this construction, a node has at most one robust edge on each end.

A mutually robust edge is defined as one that is robust going in both directions between the two nodes it connects.

Contigs are the connected components of the subgraph consisting of mutually robust edges. Singleton and circular contigs are reported for diagnostic purposes, but ignored in subsequent analysis. Each retained “*k*-mer contig” of Table 1 therefore represents a chain of nodes for SNPmers and unique *k*-mers not shared with other contigs.

After assembly of *k*-mer contigs, we connect them in longer structures using the Bambus scaffolder [26]. Because the contigs do not contain detailed read information, we map templates (read pairs) to contigs based on shared *k*-mer content, and dividing the resulting graph of contigs linked by templates into batches small enough for Bambus to process. Batches are divided so that no contigs in different batches share no templates.

We present templates linking contigs to Bambus using AMOS format [46] for the reads (template ends) mapped to each contig. Reads are included only for the contig with which it shares the most *k*-mers, if the span of those *k*-mers is ≥ *k* and the other read-end of the template similarly qualifies in a different contig. Bambus infers links between contigs by matching template identifiers shared by reads in different “linkable contigs”, then produces scaffolds as chains of contigs that are linked with consistent order and orientation.

Contiguous consensus representations for *k*-mer contigs and scaffolds were generated in two phases. In the first phase, sequence spanned by selected SNPmers and subset *k*-mers (see sections above) are joined together, separated by a number of Ns corresponding to the number of bases not spanned by *k*-mers in the subset. In the second phase, a single pass is made through the read data set, and stretches of Ns that are spanned by single reads are replaced by the sequence of the read.

### SNP phase and genotype inference

Each scaffold of the *k*-mer assembly constitutes a candidate marker for mapping. While the depth of sequence coverage on each member of the mapping panel is too low (~1X) to directly infer the genotype of individual members of the mapping pannel at individual SNPs, the tight linkage between SNPs within markers means that learning a sample’s genotype at any one reveals it at the others, effectively amplifying the sequence coverage by a factor proportional to the number of SNPs within the marker. This is the same principle exploited in genotype by sequencing (GBS) approaches to genetic mapping in the presence of reference genomes, for example in recombinant inbred lines of reference rice strains [16], and in crosses between *Drosophila* species with sequenced genomes Here we genotype offspring in the context of a cross between two outbred individuals, simultaneously inferring the phases of the SNPs (i.e., which bases appears on each of the four parental chromosomes in the cross). While the data will be insufficient to infer genotypes at many markers, all those where confident inferences can be made can be used to build the linkage map.

For the purposes of genotype inference, a marker is treated as a collection of *m* SNPs (indexed in the following by *i*∈{1,2,…,*m*}), that have been inferred to be closely linked on the genome via the *k*-mer assembly step. If the four parental chromosomes are labeled *a*,*b* in one parent and *c*,*d* in the other, then the genotyping problem is to infer which of the four possible segregation states or genotypes *ac*,*ad*,*bc*,*bd* describes each sample at each marker locus. We index samples with *j*, and denote a sample genotype by *g*_*j*_.

We assume that markers are very small compared to a chromosome, and ignore the possibility of a recombination event within individual markers. The data used for inference of the offspring genotypes consist of the number of reads from each barcoded sample *j* showing each of the four possible DNA bases *b* at each variable SNP position *i*, which we denote $n_{\mathrm{ij}}^{b}$.

If the phase *ϕ*_*i*_ of SNP *i* were known, *i.e.* which base is present in each of the four parental chromosomes, then a choice of genotype *g*_*j*_ implies a specific homozygous or heterozygous state *s*_*ij*_ ∈ *S* = {*AA*, *CC*, *TT*, *GG*, *AC*, *AT*, *AG*, *CT*, *CG*, *TG*} for SNP *i* in sample *j*. For a given phase and genotype, the likelihood function for a given SNP position in a given sample is given by either a binomial (for homozygous states) or trinomial probability mass function of the read counts, base-calling error rate ∈, and the site genotype *s*_*ij*_:

$$\begin{array}{l} L\left(\phi_{i},g_{j}\right)=p\left(n_{\mathrm{ij}}^{b}|\left|\phi_{i},g_{j}\right)\right)=P_{m}\left(n_{\mathrm{ij}}^{b}|\left|s_{\mathrm{ij}},∊\right)\right)\\ \;=\left\{\begin{array}{c} \left(\begin{array}{c} n\\ m \end{array}\right){∊}^{m}{\left(1-∊\right)}^{n-m},\;(\mathrm{if}\;s_{\mathrm{ij}}\;\mathrm{homozygous})\\ \frac{n!}{k!l!m!}{\left(\frac{2∊}{3}\right)}^{m}{\left(\frac{1-\left(\frac{2∊}{3}\right)}{2}\right)}^{k+l},\left(\mathrm{if}\;s_{\mathrm{ij}}\;\mathrm{heterozygous}\right) \end{array}\right) \end{array}$$

where *n* is the total number of reads at SNP *i*; *m* is the number of observations of bases not in *σ*_*ij*_ (i.e., mismatches); *k* and *l* are the counts for each of the two bases of *σ*_*ij*_ for heterozygous sites.

### Likelihood maximization

Searching for an optimal choice of SNP phases *ϕ*_*i*_ and sample genotypes *g*_*j*_ is made difficult by the exponential size of the search space: for segregating bi-allelic SNP sites there are 14 possible phases to consider at each SNP site, so for a mapping panel of only 20 siblings and a marker containing only 10 SNPs, there are combinations to consider. In simulation tests, we found that a variant of expectation maximization (EM), an iterative likelihood maximization method can accurately infer a large proportion of marker genotypes.

To initialize the iteration, the parental samples and a randomly selected offspring are, without loss of generality, assigned genotypes (*a*,*b*), (*c*,*d*) and (*a*,*c*). At each step, we calculate the conditional probability distributions over the possible SNP phases *p*(*ϕ*_*i*_) given the genotype assignments according to:

$$p^{\left(t\right)}\left(\phi_{i}\right)=p\left(\phi_{i}|g^{\left(t\right)}\right)=\prod_{\sigma \in S}p\left(n_{\mathrm{i\sigma}}^{b}|\phi_{i},g^{\left(t\right)}\right)/\left(\sum_{k}\prod_{\tau \in S}p\left(n_{\mathrm{k\tau}}^{b}|\phi_{k},g^{\left(t\right)}\right)\right)$$

where we have labeled the chosen values for the genotypes at iteration *t* collectively by *g*^(*t*)^*g*^(*t*)^$n_{\mathrm{i\sigma}}^{b}\;$ is the combined total number of observations of base *b* at polymorphic SNP *i* for all samples included at iteration step *t* which have genotype *s*(*ϕ*_*i*_, *g*_*j*_) = *σ*.

On each iteration until all samples have been included, a randomly selected sample is added to the set after calculating *p*^(*t*)^(*ϕ*_*i*_). Then the next set of genotype assignments *g*^(*t* + 1)^ are determined by choosing those that maximize the expected value of the log likelihood:

$$E_{\phi |n,g_{j}}\left[\log L\left(g_{j};n,\phi \right)\right]=\sum_{i}p\left(\phi_{i}\right)\log p\left(n_{\mathrm{ij}}^{b}|g_{j},\phi_{i}\right)$$

These steps are repeated until genotypes are being selected for all samples, and the expected log likelihood stops increasing. At the end of the iteration, the likelihood-maximizing genotypes are reported, along with the log likelihood difference between the best and second best choice of genotype for each sample, which provides an indicator of the confidence in genotype call. To gauge convergence, this procedure is repeated 5 times for each marker, with different random choices of initial conditions. Markers which do not identify the same ML genotype multiple times in independent runs are not included among the high confidence genotype calls.

### Map bins

Unique marker segregation patterns were included in the set of map bins if they met one of two criteria: (1) at least three independent markers were inferred to have the pattern independently, or (2) the pattern was inferred from at least one marker with at least 20 SNPs such that the mean of the estimated probabilities of the inferred SNP phases was greater than 0.9.

### Error model calibration

The sequence of 14 *Ciona intestinalis* autosomes were downloaded from Ensembl [55]. These 14 chromosomes were used as the template in our genome simulation. Based on their sequence length, We used a markovian coalescent simulator macs [56] to generate four haploid samples drawn from a population under neutral Wright-Fisher model with population mutation rate of 0.012 and population recombination rate of 0.0085. Using the *C. intestinalis* genome as the reference sequence, two diploid parental genomes were constructed based on the macs output with realistic SNP and Indel models inferred by several previous studies on the *Ciona* genome [57-59]. We wrote a perl script to simulate the genomes of offspring generated by the cross of the two simulated parents. The software package dwgsim [60] was used to generate Illumina paired-end reads based on our simulated genomes of both parents and offsprings, with the coverage of 20X and 5X respectively.

To estimate the frequency of incorrect genotype calls as a function of the log likelihood difference between the called and alternative genotype, including contributions from uncertainty in SNP-mer identification, assembly, and sampling noise, we carried out a simulation of the *k*-mer assembly and genotype inference protocols Among high-confidence genotype calls, the observed error frequency was a function of call confidence score was well-fit by a sum of two stretched exponential functions, allowing assignment of error probabilities to individual genotype calls.

### Linkage group construction

We use the linkage *p*-value *p*_*ab*_ between pairs of map bins *a* and *b* defined as the minimum over the four possible relabelings *r* of the maternal and paternal chromosomes of the Binomial *p*-value for the number of matching genotypes:

$$p_{\mathrm{ab}}=\underset{r}{\text{min}}\left[1-\sum_{i}^{m_{r}-1}\left(\begin{array}{c} n\\ i \end{array}\right)\frac{1}{2^{n}}\right]$$

where *n* is the total number of sample genotype calls (68 in the present case, or 34 in each parent) and *m*_*r*_ is the number of matching genotypes under relabeling *r*.

We identified map bins with segregation patterns indicating either inconsistent placement in the maternal and paternal maps or genotyping error with a double threshold procedure as follows:

1. Map bins were partitioned into linkage groups by single linkage clustering at a threshold of . *p*_*ab*_  < *p*_1_.

2. Within each partition, map bins which formed articulation points(i.e., nodes which, if removed, would cause the linkage group to fall apart into two disconnected subgraphs;) in the graph of, *p*_*ab*_  < *p*_2_, where *p*_2_ > *p*_1_.

This procedure identifies map bins which alone account for the merging of what would otherwise be two distinct partitions. We used the following pairs of thresholds *p*_1_, *p*_2_  to identify a total of 20 map bins for exclusion from the map: 10,10; 10,10; 10,10. The remaining markers form locally consistent linkage groups in which all linkages defined at threshold *p*_1_ are corroborated by multiple linkages at *p*_2_, for the above values of *p*_1_ and *p*_2_.

### Marker ordering

Markers were ordered within each linkage group using the following protocol. Within each linkage group a consistent labeling of the four parental chromosomes was achieved by constructing a graph *G* in which nodes correspond to map bins and edges are weighted by linkage *p*-value *p*_*ab*_ (as defined above). The local chromosome labels are updated at each map bin as it is reached in a traversal of the minimum spanning tree of *G* to the labeling *r* that maximizes *p*_*ab*_ along the incident of *G* used in the traversal. Markers within each linkage group were clustered by hierarchical clustering (marker-marker distance metric: cosine of the angle between the vectors of recombination distances to the other map bins; distance updating method: average linkage) into a binary tree data structure with leaves representing map bins. A node in the right subtree of the root node was rotated, interchanging its left and right subtrees if its left subtree was not already closer (in average recombination distance) to the markers of the left subtree of the global root; and similarly for nodes in the left subtree of the root. An in-order traversal of the tree generates an ordering of the markers. Finally three reversals of the order of markers in segments of the map were added based on visual inspection of the recombination distance matrix. In the final marker ordering, 51% of adjacent map bin pairs are separated by a single recombination event in the cross, and 94% are separated by three or fewer recombinants in each parent.

### Placement of markers on the map

To anchor additional markers to the map, we computed the *p*_*ab*_ (see above) between marker *a* to be placed on the map and each map bin *b*. Marker *a* is anchored to the map at the position of the bin *b* which minimizes *p*_*ab*_ if *p*_*ab*_  < 10^− 6^.

### SNP density estimation

Illumina reads were mapped to the assembled scaffold sequences with stampy [61] using default settings. For a sample of 9,228 scaffolds with lengths ranging from 5.0-5.5 kb, sequence variants were called with SAMtools [62] using a variant quality score threshold of 50, and ignoring indel positions.

A SNP density of 0.76% in four haplotypes corresponds to a predicted rate of pairwise sequence differences per site of Θ = 0.0042 under the finite sites model of mutation and the neutral coalescent model of the relationships among sampled alleles [63].

### Estimation of local recombination rate

To estimate the local recombination rate for each map bin, we computed the linear regression of map distance in number of markers on physical distance using up to 10 neighboring map bins in each direction along the map (or fewer for bins within 10 map bins of the end of the linkage group). Map distance was calculated from recombination fraction using Haldane’s map distance $-\frac{1}{2}\log\left(1-2r\right)$[64].

### Ancestral linkage group conservation

To compare the genome organization in *L. polyphemus* to the ancestral metazoan ALGs, we used the reciprocal best blast hit (RBH) orthology criterion in an alignment of the *Ixodes scapularis* predicted proteins [36] to the consensus sequences for the marker scaffolds. *L. polyphemus* scaffolds with RBH of e-value were assigned to the same ancestral bilaterian gene orthology group as their *I. scapularis* ortholog, and thereby with human genes. Regions of the map were tested for enrichment in genes from particular ancestral linkage groups with Fisher’s Exact Test, and breakpoints in ancestral linkage group composition were identified using a hidden Markov model, as previously described [6,7].

### Homeobox gene modeling

We identified 155 marker scaffolds with a tblastx alignment of e-value to a set of chelicerate homeobox gene sequences downloaded from Genbank using the NCBI online query interface (genbank accessions AF071402.1, AF071403.1, AF071405.1, AF071406.1, AF071407.1, AF085352.1, AF151986.1, AF151987.1, AF151988.1, AF151989.1, AF151990.1, AF151991.1, AF151992.1, AF151993.1, AF151994.1, AF151995.1, AF151996.1, AF151997.1, AF151998.1, AF151999.1, AF152000.1, AF237818.1, AJ005643.1, AJ007431.1, AJ007432.1, AJ007433.1, AJ007434.1, AJ007435.1, AJ007436.1, AJ007437.1, AM419029.1, AM419030.1, AM419031.1, AM419032.1, DQ315728.1, DQ315729.1, DQ315730.1, DQ315731.1, DQ315732.1, DQ315733.1, DQ315734.1, DQ315735.1, DQ315736.1, DQ315737.1, DQ315738.1, DQ315739.1, DQ315740.1, DQ315741.1, DQ315742.1, DQ315743.1, DQ315744.1, EU870887.1, EU870888.1, EU870889.1, HE608680.1, HE608681.1, HE608682.1, HE805493.1, HE805494.1, HE805495.1, HE805496.1, HE805497.1, HE805498.1, HE805499.1, HE805500.1, HE805501.1, HE805502.1, S70005.1, S70006.1, S70008.1, and S70010.1). The reads of each marker (those with best stampy [61] alignment to the scaffold) were reassembled with PHRAP [65], with default parameters. The resulting contigs were aligned to a collection of homeobox-containing protein sequences (genbank accessions NP 001034497.1, NP 001034510.1, AAL71874.1, NP 001034505.1, NP 001036813.1, CAA66399.1, NP 001107762.1, NP 001107807.1, EEZ99256.1, NP 001034519.1, NP 476954.1, NP 032840.1, NP 031699.2, AAI37770.1, EEN68949.1, NP 523700.2, NP 001034511.2, AAK16421.1, and AAK16422.1) with exonerate [66] in protein-to-genome mode. For each contig, the amino acid sequence predicted by the highest-scoring exonerate alignment was used in subsequent phylogenetic analysis, resulting in 104 putative homeobox-containing markers ranging in length from 18 to 147 amino acids.

### Phylogenetic analysis of homeobox genes

**Figure 8:**
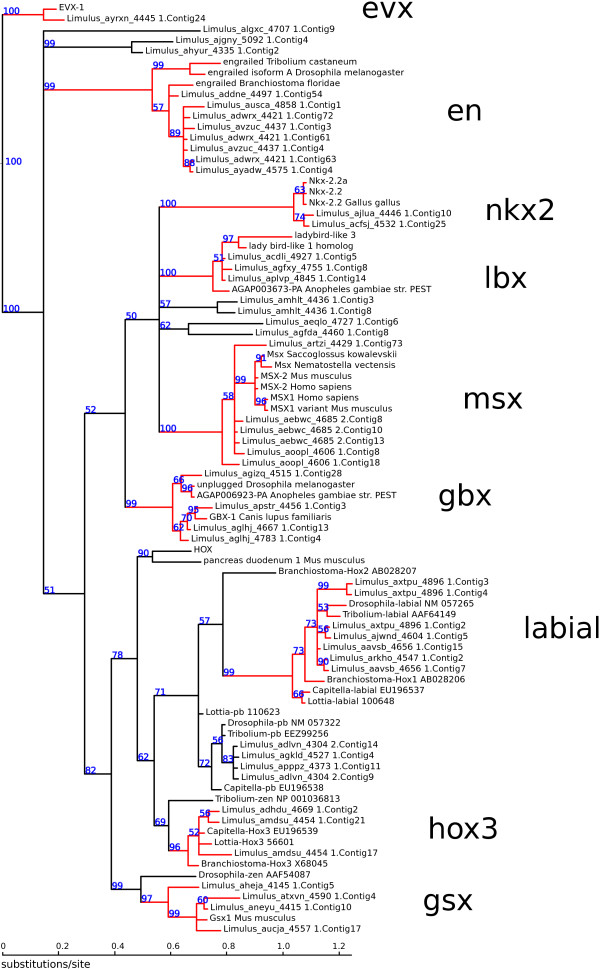
**Unrooted phylogenetic tree of homeobox sequences (part 1).** Nodes are labeled with Bayesian posterior probabilities. Highly supported partitions used to classify *L. polyphemus* sequences are drawn in red, with the abbreviation for the class shown in large letters. *L. polyphemus* homeobox sequences not grouped into one of these highly supported partitions are assigned to class “? ”. For ease of display, a large subtree consisting of HOX and parahox genes has been pruned at the position labeled “HOX”, and is shown in Figure 7.

**Figure 9:**
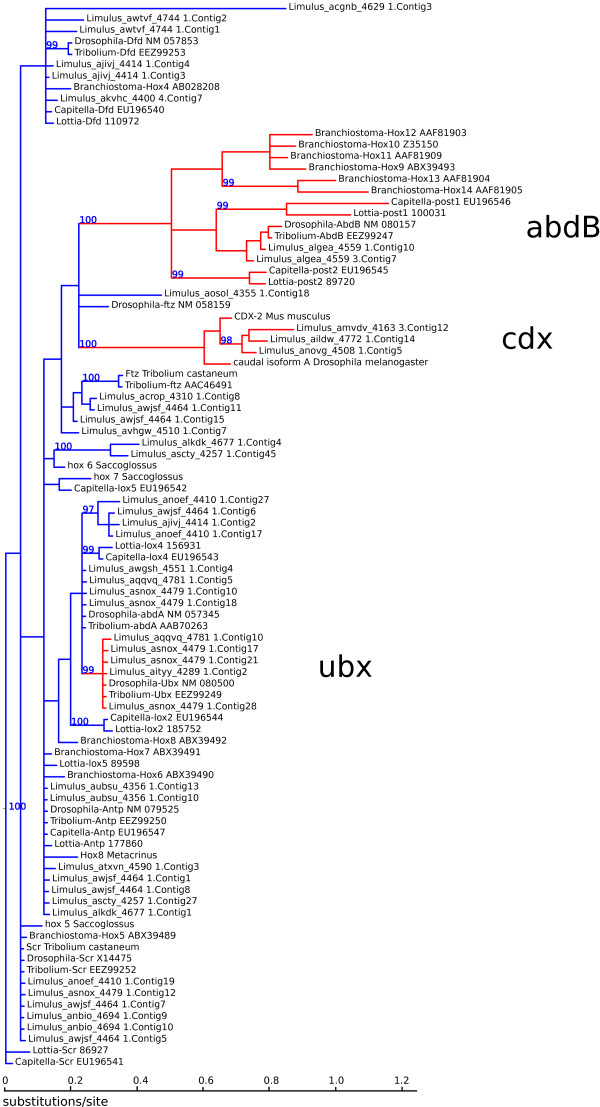
**Phylogenetic tree of homeobox sequences, part 2.** The rooted subtree pruned from the tree in Figure 8**.** Nodes are labeled with Bayesian posterior probabilities. Highly supported partitions used to classify *L. polyphemus* sequences are drawn in red, with the abbreviation for the class shown in large letters. *L. polyphemus* homeobox sequences not grouped into one of these highly supported partitions are assigned to class “hox?”.

A multiple sequence alignment of the predicted homeobox sequences combined with a collection of representative sequences from various classes of homeobox genes was constructed with muscle v3.8.31 [67] using default settings. The resulting alignment was trimmed to a 63 amino acid segment spanning the conserved homeodomain, and sequences with more than 50% gaps were removed, leaving 93 predicted *L. polyphemus* homeobox genes in the analysis. Bayesian phylogenetic analysis was carried out on the resulting 178 taxon, 63 amino acid character matrix (See Additional file 1) using MrBayes v3.2.1 [68] using a mixed model of amino acid substituions, gamma-distributed rate variation among sites with fixed shape parameter α = 1.0, alignment gaps treated as missing data, 2,000,000 Monte Carlo steps, two independent runs with four Monte Carlo chains, and the initial 25% of sampled trees were discarded as “burn-in”. Monte Carlo appeared to reach convergence, with an average standard deviation of the split frequencies of 0.022. The majority-rule consensus of the sampled trees is shown in Figures 8 and 9, and well-supported gene clades (posterior probability greater than 0.95) were used to group the predicted *L. polyphemus* genes into classes. The table in Additional file 1 lists the reassembled marker contigs, their inferred hox gene class, and maximum likelihood map positions. Predicted genes were anchored to the map as described above.

### Genomic distribution of paralogs

We identified 2,716 pairs of *Limulus* markers that can both be placed on the map and have their best translated alignment to the same *Ixodes scapularis* gene. (*I. scapularis* genes with more than five best-hit markers were excluded from seeding such pairs.) To estimate the synonymous sequence divergence between pairs of candidate *L. polyphemus* paralogous gene pairs and *L. polyphemus* genes and their *T. tridentatus* orthologs, we constructed codon alignments of predicted coding sequence for estimation of synonymous sequence divergence. Conserved clusters of paralogs were identified using a variant of the “max-gap” criterion [3] in which two genes are placed in the same cluster if they and their paralogs lie within threshold distance.

### K_a_ and K_s_ estimation for paralogs and T. tridentatus orthologs

Figure 6 shows the distribution across the map of pairs of candidate paralogs. To estimate the synonymous sequence divergence between pairs of candidate *L. polyphemus* paralogous gene pairs, and between *L. polyphemus* genes and their *T. tridentatus* orthologs, we followed the following protocol.

1. Reassemble reads from each marker with PHRAP [65] , and create a predicted coding sequence using exonerate, as described for the annotation of homeobox gene models (see above).

2. Combine the exonerate alignments of codons to amino acids to create an alignment of codons for either a pair of *L. polyphemus* sequences, or for a *L. polyphemus* - *T. tridentatus* sequence pair.

3. Use the method of Yang and Nielsen [69] to estimate the synonymous and non-synonymous substitution rates K_a_ and K_s_, as implemented in the KaKsCalculator package [70].

4. Discard estimates based on fewer than 30 sites (30 synonymous sites for estimates of K_s_, non-synonymous sites for K_a_).

GenBank accessions for *Tachypleus tridentatus* mRNA clones: JQ966943, AB353281, AB353280, HM156111, HQ221882, HQ221883, HQ221881, HQ386702, HM852953, TATTPP, TATPROCLOT, FN582225, FN582226, AF467804, AF227150, GQ260127, AF264067, AF264068, AB353279, AB005542, TATLICI, TATTGL, TATCFGB, TATLFC1, TATLFC2, AB201713, TATCFGA, TATLICI2, CS423581, CS423579, AB028144, AB201778, AB201776, AB201774, AB201772, AB201770, AB201768, AB201766, AB201779, AB201777, AB201775, AB201773, AB201771, AB201769, AB201767, AB201765, AB105059, AB002814, AX763473, TATCFBP, AB076186, AB076185, X04192, TATHCLL, AB037394, AB019116, AB019114, AB019112, AB019110, AB019108, AB019106, AB019104, AB019102, AB019100, AB019098, AB019096, AB019117, AB019115, AB019113, AB019111, AB019109, AB019107, AB019105, AB019103, AB019101, AB019099, AB019097, AB023783, AB024738, AB024739, AB024737, AB017484, D87214, D85756, D85341.

**Figure 10:**
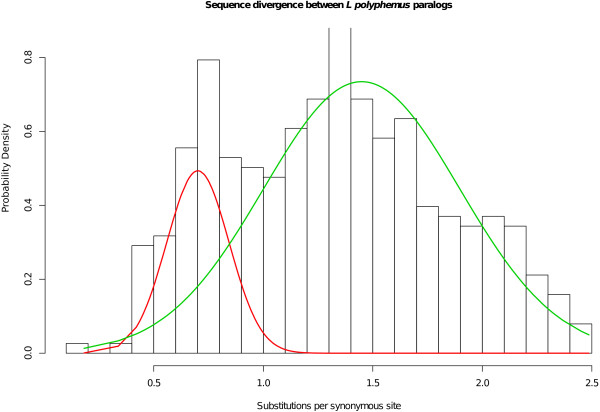
**Two component mixture model fit to the K**_**s**_**peak on the range 0  < =K**_**s**_ ** < =2.5.** The best-fitting model was selected by the Bayesian Information Criterion (Table 3). The component means are 0.7 and 1.45 substitutions per site. The position of the peak at lowest K_s_ was not sensitive to the addition of more mixture components.

Figure 7 shows the distribution of K_a_ and K_s_ for paralogs and *T. tridentatus* orthologs. To estimate the number and age of peaks in the un-saturated range [37] of the K_s_ distribution (and of putative WGD events), we fit a series of univariate normal mixture models, with 1, 2, 3, and 4 components to the paralog K_s_ distribution in the range 0  < = K_s_  < =2.5 and selected the best model on the basis of Bayesian Information Criterion (BIC) (Table 3). The best model had two components, with means at 0.7 and 1.45 substitutions per site. The position of the peak at lowest K_s_ was not sensitive to the addition of more mixture components. Figure 10 shows comparison of the distribution and the components of the best-fitting model. Gaussian mixture models were estimated in R with mixtools [71].

**Table 3:** **Mixture model fits to K**_**s **_**distribution**

**N**	**k**	**ln(L)**	**BIC**	**AIC**	**Mixture components**
1	2	−273.93	559.74	551.87	1.32 ± 0.50
2	5	−259.32	**548.31**	528.64	0.70 ± 0.14 ; 1.45 ± 0.45
3	8	−253.36	554.19	522.71	0.71 ± 0.17 ; 1.34 ± 0.29 ; 2.09 ± 0.19
4	11	−251.41	568.11	524.82	0.74 ± 0.18 ; 1.34 ± 0.20 ; 1.70 ± 0.04 ; 2.02 ± 0.22

N is the number of mixture components, k the number model parameters, ln(L) the log likelihood of the data under the best fit model.

BIC, Bayesian information criterion; AIC,Akaike information criterion.

The BIC score of our selected model is shown in bold.

## Availability of supporting data

The raw sequencing reads are currently being submitted through the NCBI SRA and are accessible via NCBI BioProject accession PRJNA187356.

The data sets supporting the results of this article are available in the *GigaScience* GigaDB repository [72].

## Abbreviations

ALG: Ancestral linkage groups; AIC: Akaike information criterion; BIC: Bayesian information criterion; bp: Basepair; EM: Expectation maximization; GBS: Genotyping-by-sequencing; JAM: Joint assembly and mapping; LG: Linkage group; Max-gap: Maximum gap; Mb: Megabase; MYA: Million years ago; PE: Paired-end; RAD-Seq: Restriction site associated DNA sequencing; RBH: Reciprocal best blast hit; SNP: Single nucleotide polymorphism; WGD: Whole-genome duplication.

## Competing interests

The authors declare that they have no competing interests.

## Authors’ contributions

NHP conceived and led the project. All authors wrote the paper. PH, NHP and J-XY wrote software. NHP, PH, J-XY and CWN carried out sequence analysis. JB collected and raised samples. CWN extracted genomic DNA and created the libraries for sequencing. All authors read and approved the final manuscript.

## Supplementary Material

Additional file 1 The amino acid character matrix used for the phylogenetic analysis of homeobox genes. Click here for file
