# Supplementary material for: Joint assembly and genetic mapping of the Atlantic horseshoe crab genome reveals ancient whole genome duplication
Source: Gigascience. 2014 May 14;3:9. doi: 10.1186/2047-217X-3-9 (PMC4066314; doi:10.1186/2047-217X-3-9)
Supplement: Additional file 1 — The amino acid character matrix used for the phylogenetic analysis of homeobox genes. [file 2047-217X-3-9-S1.pdf]

| name | sequence<br>'.' for match | accession | position<br>LG:coord. | class |
|---|---|---|---|---|
| EVX-1 | QMRRYRTAFTREQIARLEKEFYRENYVSRPRRCELAAALNLPETTIKVWFQNRRMKDKRQRLA | P23683.1 | | |
| g_ayrxn_scaff_4445_1.Contig24 | --...........L.......C..S..................S....R.------------- | | 1 : 7968 | evx |
| g_algxc_scaff_4707_1.Contig9 | KE..S.....E..LEY..RT.E.TQ.PDVYT.E...QRTK.T.ARVQ.--------------- | | 1 : 2335 | ? |
| g_ajgny_scaff_5092_1.Contig4 | KAK.I..I..A..LN...A..LQQQ.MVGRE.SH..SE...K.AQV........I.WRK---- | | 4 : 26846 | ? |
| g_ahyur_scaff_4335_1.Contig2 | -AK.V..I..A..LD...T..E.QQ.MVG.E.LQ..S....T.AQV........I.WRK.HFD | | 1 : 3106 | ? |
| engrailed__Tribolium_castaneum_ | EEK.P....SGA.L...KH..AENR.LTER..QQ.S.E.G.N.AQ..I....K.A.I.KASGQ | NP_001034511.2 | | |
| engrailed__isoform_A__Drosophila_melanogaster_ | DEK.P....SS..L...KR..NENR.LTER..QQ.SSE.G.N.AQ..I....K.A.I.KSTGS | NP_523700.2 | | |
| engrailed__Branchiostoma_floridae_ | EEK.P.....S..LQ..K...QENR.LTEQ..QD..RE.K.N.SQ..I....K.A.I.KAAGV | EEN68949.1 | | |
| g_addne_scaff_4497_1.Contig54 | EEK.P.....TD.L...K...HENR.LTEK..Q...RE.Q.N.SQ..I....K.A.I.KSTGE | | 1 : 5794 | en |
| g_ausca_scaff_4858_1.Contig1 | DEK.P.....AD.L...KQ..QENR.LTEK..QD..LD.Q.N.SQ..I....K.A.I.KASGQ | | 20 : 83001 | en |
| g_adwrx_scaff_4421_1.Contig72 | DEK.P.....AD.L...KA..QENR.LTEK..QD..RG.Q.N.SQ..I....K.A.I.KATGH | | 15 : 71297 | en |
| g_avzuc_scaff_4437_1.Contig3 | DEK.P.....TD.L...KA..QENK-LTEK..QD..RE------------------------- | | 21 : 85367 | en |
| g_adwrx_scaff_4421_1.Contig61 | DEK.P.....TD.L...KA..QENR.LTEK..QD..RE.Q.N.SQ..I.-------------- | | 15 : 71297 | en |
| g_avzuc_scaff_4437_1.Contig4 | -------------------..QENR.LTEK..QD..RE.Q.N.SQ..I....K.A.I.KATGH | | 21 : 85367 | en |
| g_adwrx_scaff_4421_1.Contig63 | -EK.P.....AD.L...KA..QENR.LTEK..QD..RE.Q.N.SQ..I....K.A.I.KAVGH | | 15 : 71297 | en |
| g_ayadw_scaff_4575_1.Contig4 | DEK.P.....AD.L...KA..QENR.LTEK..QD..RE.Q.N.SQ..I....K.A.I.KAVGH | | 3 : 22654 | en |
| Nkx-2.2a | KK.KR.VL.SKA.TYE..RR.RQQR.L.A.E.EH..SLIR.TP.QV.I....H.Y.M..A.AE | NP_571497.1 | | |
| Nkx-2.2 | KK.KR.VL.SKA.TYE..RR.RQQR.L.A.E.EH..SLIR.TP.QV.I....H.Y.M..A.AE | NP_002500.1 | | |
| Nkx-2.2__Gallus_gallus_ | KK.KR.VL.SKA.TYE..RR.RQQR.L.A.E.EH..SLIR.TP.QV.I....H.Y.M..A.AE | XP_003643855.1 | | |
| g_ajlua_scaff_4446_1.Contig10 | KK.KR.VL.SKA.TYE..RR.RQQR.L.A.E.EH..NFIR.TP.QV.I....H.Y.T..A.QE | | 1 : 1567 | nkx2 |
| g_acfsj_scaff_4532_1.Contig25 | KK.KR.VL.SKA.TYE..RR.RQQR.L.A.E.EH..SIIR.TP.QV.I....H.Y.T..A.QE | | 3 : 20058 | nkx2 |
| ladybird-like__3 | KR.KS....SNQ.LFE..RR.V.QK.L.PAD.DQ..QR.A.SSAQVIT......A.L..DLDE | NP_001038133.1 | | |
| lady_bird-like__1_homolog | KR.KS.....NH..YE...R.LYQK.L.PAD.DQI.QQ.G.TNAQVIT......A.L..DLEE | NP_001007135.1 | | |
| g_acdli_scaff_4927_1.Contig5 | KK.KS.....NH..FE...R.LYQK.L.PAD.D.I.QS.G.TNAQVIT......A.L..---- | | 26 : 93066 | lbx |
| g_agfxy_scaff_4755_1.Contig8 | KK.KS.....NQ..FE...R.LYQK.L.PAD.D.I.QS.R.TNAQVIT......A.L..---- | | 13 : 67147 | lbx |
| g_aplvp_scaff_4845_1.Contig14 | KK.KS.....NH..FE...R.LYQK.L.PAD.D.I.QS.G.TNAQVIT......A.L..---- | | 1 : 3106 | lbx |
| AGAP003673-PA__Anopheles_gambiae_str._PEST_ | KK.KS.....NH..FE...R.LYQK.L.PSD.D.I....G.SNAQVIT......A.L..DMEE | EAA04094 | | |
| g_amhlt_scaff_4436_1.Contig3 | KQ.KA.....DH.LQT...C.E.QK.L.VQE.M...SK...TD.QV.T.Y...---------- | | 8 : 46729 | ? |
| g_amhlt_scaff_4436_1.Contig8 | KE.KA.....DY..QT...R.QTQK.L.VQD.M.I.SK...TD.HV.T.Y...---------- | | 8 : 46729 | ? |
| g_aeqlo_scaff_4727_1.Contig6 | RKKKT..V...S.VFQ..ST.DMKR.L.SSE.AG...C.H.T..QV.I......N.W...--- | | 22 : 86973 | ? |
| g_agfda_scaff_4460_1.Contig8 | KRKKS..T..GR..FE...Q.EIKK.L.SSE.A.M.KL..VT.QQVEI--------------- | | 26 : 93149 | ? |
| g_artzi_scaff_4429_1.Contig73 | SN.KL..P..TQ.LLA..RK.LAKQ.L.IAE.AKFSIS.T.T.AQV.I......A.E..---- | | 5 : 32215 | msx |
| Msx__Saccoglossus_kowalevskii_ | TN.KP..P..TS.LLA..RK.RQKQ.L.IAE.A.FS.S...T..QV.I......A.A..LQE. | ABD97280 | | |
| Msx___Nematostella_vectensis_ | AN.KP..P..TS.LLA..RK.RQKQ.L.IAE.A.FS.S...T..QV.I......A.A..LHE. | BAG11598 | | |
| MSX-2__Mus_musculus_ | TN.KP..P..TS.LLA..RK.RQKQ.L.IAE.A.FSSS...T..QV.I......A.A..LQE. | NP_038629 | | |
| MSX-2__Homo_sapiens_ | TN.KP..P..TS.LLA..RK.RQKQ.L.IAE.A.FSSS...T..QV.I......A.A..LQE. | BAA06549 | | |
| MSX1___Homo_sapiens_ | TN.KP..P..TA.LLA..RK.RQKQ.L.IAE.A.FSSS.S.T..QV.I......A.A..LQE. | NP_002439 | | |
| MSX1_variant__Mus_musculus_ | TN.KP..P..TA.LLA..RK.RQKQ.L.IAE.A.FSSS.S.T..QV.I......A.A..LQE. | AAG32466 | | |
| g_aebwc_scaff_4685_2.Contig8 | -----------------------KQ.L.IAE.A.FSSS.S.T..QV.I......A.E..LKE. | | 3 : 19118 | msx |
| g_aebwc_scaff_4685_2.Contig10 | SN.KP..P..TQ.LLA..RK.RTKQ.L.IAE.A.FSSS...T..QV.I......A.E..---- | | 3 : 19118 | msx |
| g_aebwc_scaff_4685_2.Contig13 | SN.KP..P..TQ.LLA..RK.RSKQ.L.IAE.A.FSSS...T..QV.I......A.E..---- | | 3 : 19118 | msx |
| g_aoopl_scaff_4606_1.Contig8 | -----------------------KQ.L.IAE.A.FSSS...T..QV.I......A.EN.LKE- | | 26 : 93149 | msx |
| g_aoopl_scaff_4606_1.Contig18 | SS.KP..P..TK.LLA...M.QTKQ.L.IAE.A.FSSSM..T..QV.I......A.E..---- | | 26 : 93149 | msx |
| g_agizq_scaff_4515_1.Contig28 | --..R...Y.S..LLQ....HAKK.L.LTE.SQISTT.Q.S.VQV.I......A.W..---- | | 3 : 23600 | gbx |
| unplugged__Drosophila_melanogaster_ | KS..R.....S..LLE..R..HAKK.L.LTE.SQI.TS.K.S.VQV.I......A.W..VKAG | NP_477146 | | |
| AGAP006923-PA__Anopheles_gambiae_str._PEST_ | KS..R.....S..LLE..R..HAKK.L.LTE.SQI.TS.K.S.VQV.I......A.W..VKAG | EAA04094 | | |
| g_apstr_scaff_4456_1.Contig3 | --..R.....N..LLE..N..HCKK.L.LTE.SQI.HS.Q.S.QQV.I......A.W..---- | | 1 : 8283 | gbx |
| GBX-1__Canis_lupus_familiaris_ | KS..R.....S..LLE....HCKK.L.LTE.SQI.H..K.S.VQV.I......A.W..IKAG | XP_853664 | | |

| name | sequence<br>'.' for match | accession | position<br>LG:coord. | class |
| --- | --- | --- | --- | --- |
| g_aglhj_scaff_4667_1.Contig13 | --..R.....S..LLE.....HSKK.L.LTE.SQI.HS.Q.S.VQV.I......A.W..---- | | 11 : 60310 | gbx |
| g_aglhj_scaff_4783_1.Contig4 | KS..R.....S..LLE.....HSKK.L.LTE.SHI.NS.Q.S.VQV.I......A.W..VKAG | | 18 : 80043 | gbx |
| g_acgnb_scaff_4629_1.Contig3 | RFLLSLSQR.SRNGSQATDGLHFNRWLT.R..I.IVRT.C.SD-------------------- | | 20 : 83755 | hox? |
| g_awtvf_scaff_4744_1.Contig2 | ETK.Q...Y..H..LE.....HFNR.LT.R..I.I.HSSC.ERIK.----------------- | | 21 : 85243 | hox? |
| g_awtvf_scaff_4744_1.Contig1 | -----------------------SR.LT.R.QI.I.HS.C.S.RQ..N...I....W.KDNKL | | 21 : 85243 | hox? |
| Drosophila-Dfd__NM_057853_ | EPK.Q...Y..H..LE.....HYNR.LT.R..I.I.HT.V.S.RQ..I........W.KD--- | NM_057853 | | |
| Tribolium-Dfd__EEZ99253_ | EPK.Q...Y..H..LE.....HYNR.LT.R..I.I.HT.V.S.RQ..I........W.KD--- | EEZ99253 | | |
| g_ajivj_scaff_4414_1.Contig4 | ETK.Q...Y..H..LE.....HFNR.LT.R..I.I.HL.C.S.RQ..I........W.KDNKL | | 15 : 71079 | hox? |
| g_ajivj_scaff_4414_1.Contig3 | ETK.Q...Y..H..LE.....HFNR.LT.R..I.I.HS.C.S.RQ..I........W.KDNKL | | 15 : 71079 | hox? |
| Branchiostoma-Hox4__AB028208_ | DTK.S...Y..Q.VLE.....HFNR.LT.R..I.I.HS.G.T.RQ..I........W.KD--- | AB028208 | | |
| g_akvhc_scaff_4400_4.Contig7 | EAK.Q...Y..H.VLE.....HFNR.LT.R..I.I.H..C.S.RQ..I........W.KDNKL | | 18 : 79524 | hox? |
| Capitella-Dfd__EU196540_ | DSK.T...Y..H..LE.....HFNR.LT.R..I.I.HT.C.S.RQ..I........W.KE--- | EU196540 | | |
| Lottia-Dfd__110972_ | DSK.N...Y..H.VLE.....HFNR.LT.R..I.I.HT.C.S.RQ..I........W.KE--- | JGI Lotgi1 protein id: 110972 | | |
| Branchiostoma-Hox12__AAF81903_ | SS.KK.CPYSKV.LLE.....LYNM.IT.EQ.G.I.RKV..TDRQV.I........M..M--- | AAF81903 | | |
| Branchiostoma-Hox10__Z35150_ | VG.KK.CPY.KY..LE.....LFNM....E..Q.ISRHV..SDRQV.I........M..M--- | Z35150 | | |
| Branchiostoma-Hox11__AAF81909_ | ST.KK.CPY.KY.TLE.....LFNMF.T.E..Q.I.RQ...TDRQV.I........M..M--- | AAF81909 | | |
| Branchiostoma-Hox9__ABX39493_ | SS.KK.CPY..F.TLE.....LYNM.LT.E..Y.ISQHV..T.RQV.I........M.KM--- | ABX39493 | | |
| Branchiostoma-Hox13__AAF81904_ | GG.KK.CPY.KY.LSV..Q.YIQNR....ET.L..SQR...TDRQV.I........Q..L--- | AAF81904 | | |
| Branchiostoma-Hox14__AAF81905_ | PV.PK.RPYSKY.LNE..N.YVQNQ.I..DK.LQ.SQK...T.RQV.I......I.Q.KL--- | AAF81905 | | |
| Capitella-post1__EU196546_ | NPKKK.KPYSKP.VSA..N.YSTST.ITKA..K.V.RE.D.T.RQ..I.Y....I.E.KI--- | EU196546 | | |
| Lottia-post1__100031_ | TL.KR.RPYSKF...E..R.YNGST...KS..W..SQLI..S.RQ..I......I.A.KI--- | JGI Lotgi1 protein id: 100031 | | |
| Drosophila-AbdB__NM_080157_ | SV.KK.KPYSKF.TLE.....LFNA...KQK.W...RN.Q.T.RQV.I........N.KN--- | NM_080157 | | |
| Tribolium-AbdB__EEZ99247_ | TV.KK.KPYSKF.TLE.....LFNA...KQK.W...RN...T.RQV.I........N.KN--- | EEZ99247 | | |
| g_algea_scaff_4559_1.Contig10 | TV.KK.KPYSKF.TLE.....LFNA...KQK.W...RN...T.RQV.I........S.KNAQR | | 15 : 71736 | abdB |
| g_algea_scaff_4559_3.Contig7 | ------KPYSKF.TLE.....LFNS...KQK.W...RN...T.RQV.I........S.KSSQR | | 21 : 85120 | abdB |
| Capitella-post2__EU196545_ | KQ.KK.KPY..Y.TMV..N..INNS.IT.QK.W.ISCK.H.S.RQV..........R.KL--- | EU196545 | | |
| Lottia-post2__89720_ | KG.KK.KPY..Y.TMV..N..LSSS.IT.QK.W.ISCK.Q.S.RQV..........R.KL--- | JGI Lotgi1 protein id: 89720 | | |
| g_aosol_scaff_4355_1.Contig18 | ---------------------EFNR.ITQR..I.I.HY.S.S.RK..M.....S..ANEKH-- | | 20 : 83145 | hox? |
| Drosophila-ftz__NM_058159_ | DSK.T.QTY..Y.TLE.....HFNR.IT.R..IDI.N..S.S.RQ..I........S.KD--- | NM_058159 | | |
| CDX-2__Mus_musculus_ | TKDK..VVY.DH.RLE.....HFSR.ITIR.KS....T.G.S.RQV.I......A.ERKIKKK | AAA19645 | | |
| g_amvdv_scaff_4163_3.Contig12 | TRDK..VVY.DQ.RLE.....HYNR.I.IR.KT....MVG.S.RQV.I......A.ER.NFRK | | 5 : 33811 | cdx |
| g_aildw_scaff_4772_1.Contig14 | TKDK...VY.DH.RLE.....HYSR.ITIR.KT...TM.G.SDRQV.I......A.ERK.ARK | | 19 : 80739 | cdx |
| g_anovg_scaff_4508_1.Contig5 | TKEK..VVY.DH.RLE......YSR.ITIR.KS....M.G.S.RQV.I......A.ERK.ARK | | 3 : 21395 | cdx |
| caudal__isoform_A__Drosophila_melanogaster_ | TKDK..VVY.DF.RLE....YCTSR.ITIR.KS...QT.S.S.RQV.I......A.ERK.NKK | NP_476954.1 | | |
| Ftz__Tribolium_castaneum_ | GNK.T.QTY..Y.TLE.....HFNK.LT.R..I.I.ES.R.T.RQ..I........A.KDTKF | AF321227_1 | | |
| Tribolium-ftz__AAC46491_ | GNK.T.QTY..Y.TLE.....HFNK.LT.R..I.I.ES.R.T.RQ..I........A.KD--- | AAC46491 | | |
| g_acrop_scaff_4310_1.Contig8 | APK.T.QTY..Y.TLE.....HFNK.LT.R..I.I.HT.G.T.RQ..I........E.KENK- | | 15 : 71825 | hox? |
| g_awjsf_scaff_4464_1.Contig11 | --K.T.QTY..Y.TLE.....HFNK.LT.R..I.I.HT.G.T.RQ..I....----------- | | 21 : 85120 | hox? |
| g_awjsf_scaff_4464_1.Contig15 | APK.T.QTY..Y.TLE.....HFNR.LT.R..I.I.HT.G.T.RQ..I........A.KENKF | | 21 : 85120 | hox? |
| g_avhgw_scaff_4510_1.Contig7 | --K.T.QTY..Y.TLE.....HFNR.LT.R..I.I.HS.G.T.RQ..I........A.KEMPK | | 18 : 79607 | hox? |
| g_alkdk_scaff_4677_1.Contig4 | DQ..C.QTYSFY.TLA.....HFDP.LTKR..I.I.HT...K.KQ..I........W.KEKRS | | 28 : 94741 | hox? |
| g_ascty_scaff_4257_1.Contig45 | DQ..S.QTYSFY.TLA.....HLDP.LTQR..I.I.HT...T.KQ.QI........W.KENKT | | 17 : 77601 | hox? |
| hox_6_Saccoglossus | -Q..G.QTY..Y.TLE.....HFNR.LT.R..I.I.HT.G.T.RQ..I........W.KEQK- | ABK00020 | | |
| hox_7_Saccoglossus | -KK.C.QTY..Y.TLE.....HYNR.LT.R..I..SHL.G.T.RQ..I........Y.KESK- | AAP79287 | | |
| Capitella-lox5__EU196542_ | EQK.T.QTY..Y.TLE.....HYNR.LT.R..I.I.H..Q.T.RQ..I........Y.KE--- | EU196542 | | |
| g_anoef_scaff_4410_1.Contig27 | PR..G.QSY..F.TLE.....HFNH.LT.R..I...HVVC.T.RQ..I........L.KEVRV | | 3 : 23600 | hox? |

| name | sequence | accession | position | class |
|---|---|---|---|---|
| | '.' for match | | LG:coord. | |
| g_awjsf_scaff_4464_1.Contig6 | ----G.Q.Y..F.TLE....HYNH.LT.R..I.I.HVVC.T--------------------- | | 21 : 85120 | hox? |
| g_ajivj_scaff_4414_1.Contig2 | ---.S.Q.Y..F.TLE....HYNH.LT.R..I.I.HVVC.T--------------------- | | 15 : 71079 | hox? |
| g_anoef_scaff_4410_1.Contig17 | --------------LE....HYNH.LT.R..I.I.HVVC.T.RQ..I........L.KEIR. | | 3 : 23600 | hox? |
| Lottia-lox4__156931_ | .R..G.QTYS.Y.TLE....QFNH.LT.K..I.I.HT.C.T.RQ..I........M.KE--- | JGI Lotgi1 protein id: 156931 | | |
| Capitella-lox4__EU196543_ | .R..G.QTYS.Y.TLE....QFNH.LT.K..I.I.H..C.T.RQ..I........L.KE--- | EU196543 | | |
| g_awgsh_scaff_4551_1.Contig4 | -R..G.QTY..F.TLE....HFNH.LT.R..I.I.H..C.T.RQ..I........L.KEIR. | | 15 : 71699 | hox? |
| g_aqqvq_scaff_4781_1.Contig5 | -----------------...HFNH.LT.R..I.I.H..C.T.RQ..I........L.KEIR. | | 21 : 85040 | hox? |
| g_asnox_scaff_4479_1.Contig10 | --------------------HFNH.LT.R..I.I.H..C.T.RQ..I........L.KEIR. | | 15 : 71793 | hox? |
| g_asnox_scaff_4479_1.Contig18 | --------------------HFNH.LT.R..I.I.H..C.T.RQ..I........L.KEIR. | | 15 : 71793 | hox? |
| Drosophila-abdA__NM_057345_ | PR..G.QTY..F.TLE....HFNH.LT.R..I.I.H..C.T.RQ..I........L.KE--- | NM_057345 | | |
| Tribolium-abdA__AAB70263_ | PR..G.QTY..F.TLE....HFNH.LT.R..I.I.H..C.T.RQ..I........L.KE--- | AAB70263 | | |
| g_aqqvq_scaff_4781_1.Contig10 | --------YP.Y.TLE....HTNH.LT.R..I.M.H..C.T.RQ..I........L.KEIQ. | | 21 : 85040 | ubx |
| g_asnox_scaff_4479_1.Contig17 | ------------------------.LT.R..I.M.H..C.T.RQ..I........L.KEIQ. | | 15 : 71793 | ubx |
| g_asnox_scaff_4479_1.Contig21 | LR..G.QTY..Y.TLE....HTNH.LT.R..I.M.H..C.---------------------- | | 15 : 71793 | ubx |
| g_aityy_scaff_4289_1.Contig2 | LR..G.QTY..Y.TLE....HTNH.LT.R..I.M.H..C.T.RQ..I.....---------- | | 10 : 54444 | ubx |
| Drosophila-Ubx__NM_080500_ | LR..G.QTY..Y.TLE....HTNH.LT.R..I.M.H..C.T.RQ..I........L.KE--- | NM_080500 | | |
| Tribolium-Ubx__EEZ99249_ | LR..G.QTY..Y.TLE....HTNH.LT.R..I.M.H..C.T.RQ..I........L.KE--- | EEZ99249 | | |
| g_asnox_scaff_4479_1.Contig28 | LR..G.QTY..Y.TLE....HTNH.LT.R..I.M.H..C.T.RQ..I........L.KEIQ. | | 15 : 71793 | ubx |
| Capitella-lox2__EU196544_ | .R..G.QTY..Y.TLE....KFNR.LT.R..I..SHM.C.T.RQ..I........E.KE--- | EU196544 | | |
| Lottia-lox2__185752_ | .R..G.QTY..F.TLE....KFNR.LT.R..I..SHM.C.T.RQ..I........E.KE--- | JGI Lotgi1 protein id: 185752 | | |
| Branchiostoma-Hox8__ABX39492_ | ER..G.QTYS.Y.TLE....HFNK.LT.R..I.I.H..G.T.RQ..I........L.KE--- | ABX39492 | | |
| Branchiostoma-Hox7__ABX39491_ | ERK.G.QTY..Y.TLE....HFNK.LT.R..I.I.H..C.T.RQ..I........W.KE--- | ABX39491 | | |
| Lottia-lox5__89598_ | EQK.T.QTY..Y.TLE....HFNR.LT.R..I.V.HM.G.T.RQ..I........W.KE--- | JGI Lotgi1 protein id: 89598 | | |
| Branchiostoma-Hox6__ABX39490_ | EKK.G.QTY..Y.TLE....HFNK.LT.K..I.I.HL.G.T.RQ..I........W.KE--- | ABX39490 | | |
| g_aubsu_scaff_4356_1.Contig13 | ERK.G.QTY..Y.TLE....HFNR.LT.R..I.I.H..C.T.RQ..I........W.KENK. | | 15 : 71793 | hox? |
| g_aubsu_scaff_4356_1.Contig10 | ERK.G.QTY..Y.TLE....HFNR.LT.R..I.I.H..C.T.RQ..I........W.KENK. | | 15 : 71793 | hox? |
| Drosophila-Antp__NM_079525_ | ERK.G.QTY..Y.TLE....HFNR.LT.R..I.I.H..C.T.RQ..I........W.KE--- | NM_079525 | | |
| Tribolium-Antp__EEZ99250_ | ERK.G.QTY..Y.TLE....HFNR.LT.R..I.I.H..C.T.RQ..I........W.KE--- | EEZ99250 | | |
| Capitella-Antp__EU196547_ | ERK.G.QTY..Y.TLE....HFNR.LT.R..I.I.H..C.T.RQ..I........W.KE--- | EU196547 | | |
| Lottia-Antp__177860_ | NRK.G.QTY..Y.TLE....HFNR.LT.R..I.I.H..C.T.RQ..I........W.KE--- | JGI Lotgi1 protein id: 177860 | | |
| Hox8_Metacrinus | -KK.G.QTY..Y.TLE....HFNR.LT.R..I.I.Q.VC.S.RQ..I........A.KETS- | BAF43726 | | |
| g_atxvn_scaff_4590_1.Contig3 | -------------------------LT.R..I.I.H..C.T.RQ..I........W.QENK. | | 5 : 33116 | hox? |
| g_awjsf_scaff_4464_1.Contig1 | --K.G.QTY..Y.TLE....HFNR.LT.R..I.I.--------------------------- | | 21 : 85120 | hox? |
| g_awjsf_scaff_4464_1.Contig8 | ERK.G.QTY..Y.TLE....HFNR.LT.R..I.I.H..C.T.R------------------- | | 21 : 85120 | hox? |
| g_ascty_scaff_4257_1.Contig27 | ------------.TLE....HFNR.LT.R..I.I.H..C.T.RQ..I--------------- | | 17 : 77601 | hox? |
| g_alkdk_scaff_4677_1.Contig1 | -------TY..Y.TLE....HFNR.LT.R..I.I.H..C.T--------------------- | | 28 : 94741 | hox? |
| hox_5_Saccoglossus | -AK.S...Y..Y.TLE....HFNR.LT.R..I.I.H..G.S.RQ..I........W.KEHN- | NP_001158410 | | |
| Branchiostoma-Hox5__ABX39489_ | DNK.T...Y..Y.TLE....HFNR.LT.R..I.I.H..C.T.RQ..I........W.KE--- | ABX39489 | | |
| Scr__Tribolium_castaneum_ | ETK.Q..SY..Y.TLE....HFNR.LT.R..I.I.H..C.T.RQ..I........W.KEHKM | AF321227_2 | | |
| Drosophila-Scr__X14475_ | ETK.Q..SY..Y.TLE....HFNR.LT.R..I.I.H..C.T.RQ..I........W.KE--- | X14475 | | |
| Tribolium-Scr__EEZ99252_ | ETK.Q..SY..Y.TLE....HFNR.LT.R..I.I.H..C.T.RQ..I........W.KE--- | EEZ99252 | | |
| g_anoef_scaff_4410_1.Contig19 | -----------------...HFNR.LT.R..I.I.H..C.S.RQ..I........W.KEHKM | | 3 : 23600 | hox? |
| g_asnox_scaff_4479_1.Contig12 | -------SY..Y.TLE....HFNR.LT.R..I.I.H..C.S.RQ..I--------------- | | 15 : 71793 | hox? |
| g_awjsf_scaff_4464_1.Contig7 | ETK.Q..SY..Y.TLE....HFNR.LT.R..I.I.H..C.S.RQ..I........W.KEH-- | | 21 : 85120 | hox? |
| g_anbio_scaff_4694_1.Contig9 | ETK.Q..SY..Y.TLE....HFNR.LT.R..I.I.H..C.S.RQ..I........W.KEHKM | | 15 : 71825 | hox? |

| name | sequence | accession | position | class |
|---|---|---|---|---|
| | '.' for match | | LG:coord. | |
| g_anbio_scaff_4694_1.Contig10 | ETK.Q..SY..Y.TLE.....HFNR.LT.R..I.I.H..C.S.RQ..I........W.KEHKM | | 15 : 71825 | hox? |
| g_awjsf_scaff_4464_1.Contig5 | --K.Q..SY..Y.TLE.....HFNR.LT.R..I.I.H..C.S--------------------- | | 21 : 85120 | hox? |
| Lottia-Scr__86927_ | DSK.S..SY..H.TLE.....HYNK.LT.R..I.I.H...T.RQ..I........W.KD--- | JGI Lotgi1 protein id: 86927 | | |
| Capitella-Scr__EU196541_ | DNK.T..SY..H.TLE.....HFNR.LT.R..I.I.HS..T.RQ..I........W.KE--- | EU196541 | | |
| pancreas_duodenum___1__Mus_musculus_ | ENK.T...Y..A.LLE.....LFNK.I.....V...VM...T.RH..I........W.KEEDK | NP_032840 | | |
| Branchiostoma-Hox2__AB028207_ | SS..L..V..NT.LLE.....HYNK..CK...K.I.SY.D.N.RQV.I.......RQ..R--- | AB028207 | | |
| g_axtpu_scaff_4896_1.Contig3 | ---SG..N..TK.L....N..LFNK.LT.K..I.I...IQ.N..QV.I........Q.KRMKE | | 9 : 53730 | labial |
| g_axtpu_scaff_4896_1.Contig4 | ---SG..N..TK.L.G..N..LFNK.LT.K..I.I...IQ.N..QV.---------------- | | 9 : 53730 | labial |
| Drosophila-labial__NM_057265_ | -NNSG..N..NK.LTE.....HFNR.LT.A..I.I.NT.Q.N..QV.I........Q.KRV-- | NM_057265 | | |
| Tribolium-labial__AAF64149_ | CLNTG..N..NK.LTE.....HFNK.LT.A..I.I.S..Q.N..QV.I........Q.KR--- | AAF64149 | | |
| g_axtpu_scaff_4896_1.Contig2 | --------------------------LT.A..I.I.T..Q.N..QV.I........Q.KRMKE | | 9 : 53730 | labial |
| g_ajwnd_scaff_4604_1.Contig5 | -VGSG..N..TK.LTE.....HFNK.LT.A..I.I.T..Q.N..QV.I........Q.KRMKE | | 15 : 71825 | labial |
| g_aavsb_scaff_4656_1.Contig15 | ----G..N..TK.LTE.....HFNK.LT.A..I.I.N..Q.N..QV.I........Q.KRMKE | | 18 : 79564 | labial |
| g_arkho_scaff_4547_1.Contig2 | ----G..N..TK.LTE.....HFNK.LT.A..I.I.NV.Q.N..QV.I........Q.----- | | 1 : 5456 | labial |
| g_aavsb_scaff_4656_1.Contig7 | ------------------------------..I.I.NV.Q.N..QV.I........Q.KRMKE | | 18 : 79564 | labial |
| Branchiostoma-Hox1__AB028206_ | GPNNG..N..TK.LTE.....HYNK.LT.A..V.I......N..QV.I........Q.KR--- | AB028206 | | |
| Capitella-labial__EU196537_ | -PNMG..N..NK.LTE.....HFNK.LT.A..I.I..S.G.N..QV.I........Q.KRL-- | EU196537 | | |
| Lottia-labial__100648_ | -PNSG..N..NK.LTE.....HFNK.LT.A..I.I..S.G.N..QV.I........Q.KR--- | JGI Lotgi1 protein id: 100648 | | |
| Lottia-pb__110623_ | GS..L...Y.NT.LLE.....HFNK.LC....I.I..S.D.T.RQV..........Y...--- | JGI Lotgi1 protein id: 110623 | | |
| Drosophila-pb__NM_057322_ | LP..L...Y.NT.LLE.....HFNK.LC....I.I..S.D.T.RQV..........H...--- | NM_057322 | | |
| Tribolium-pb__EEZ99256_ | LP..L...Y.NT.LLE.....HFNK.LC....I.I..S.D.T.RQV..........H...--- | EEZ99256 | | |
| g_adlvn_scaff_4304_2.Contig14 | MP..L...Y.NT.LLE.....HFNK.LC....I.I..S.D.S.RQV..........H...TMM | | 15 : 71870 | ? |
| g_agkld_scaff_4527_1.Contig4 | MP..L...Y.NT.LLE.....HFNK.LC....I.I..S.D.S.RQV..........H...TVM | | 3 : 19288 | ? |
| g_apppz_scaff_4373_1.Contig11 | MP..L...Y.NT.LLE.....HFNK.LC....I.I..S.D.S.RQV..........H...TTM | | 21 : 85040 | ? |
| g_adlvn_scaff_4304_2.Contig9 | MP..L...Y.NT.LLE.....HFNK.LC...-------------------------------- | | 15 : 71870 | ? |
| Capitella-pb__EU196538_ | HP..L...Y.NT.LLE.....HFNK.LC....I.I..S.D.T.RQV..........F...--- | EU196538 | | |
| Tribolium-zen__NP_001036813_ | AGK.A...Y.SA.LVE..R..HHGK.L.....IQI.EN...S.RQ..I........H.KE--- | NP_001036813 | | |
| g_adhdu_scaff_4669_1.Contig2 | PTK.V...Y.SA.LVE.....HFNR.LC....I.M.NL...T.RQ..I........Y.K---- | | 21 : 85075 | hox3 |
| g_amdsu_scaff_4454_1.Contig21 | --K.A...Y.SA.LVE.....HFNR.LC....V.M.NL.S.T.RQ..I........Y.KEQKN | | 15 : 71825 | hox3 |
| Capitella-Hox3__EU196539_ | PSK.A...Y.SA.LVE.....HFNR.LC....I.M..L...T.RQ..I........Y.KD--- | EU196539 | | |
| Lottia-Hox3__56601_ | PAK.A...Y.SA.LVE.....HFNR.LC....I.M..L...S.RQ------------------ | JGI Lotgi1 protein id: 56601 | | |
| g_amdsu_scaff_4454_1.Contig17 | --K.P...Y.TA.LTE.....HFTR.LC....V.M.GL.H.T.RQ..I.......-------- | | 15 : 71825 | hox3 |
| Branchiostoma-Hox3__X68045_ | AGK.A...Y.SA.LVE.....HFNR.LC....V.M..M...T.RQ..I........Y.KE--- | X68045 | | |
| Drosophila-zen__AAF54087_ | KLK.S.....SV.LVE..N..KSNM.LY.T..I.I.QR.S.C.RQV.I........F.KD--- | AAF54087 | | |
| g_aheja_scaff_4145_1.Contig5 | KTK.T..S.NGV.LLE.....TNNM.L..L..I.I.NY...S.KQV.I......V.F.KEG-- | | 23 : 89444 | gsx |
| g_atxvn_scaff_4590_1.Contig4 | SSKKI..VY..S.LLE..R..AANM.LT.L..I.I.TY.S.S.KQV.I......V.Y.KE--- | | 5 : 33116 | gsx |
| g_aneyu_scaff_4415_1.Contig10 | SSK.I.....ST.LLE..R..SANM.L..L..I.I.TY...S.KQV.I......V.Y.KE--- | | 6 : 37542 | gsx |
| Gsx1___Mus_musculus_ | SSK.M.....ST.LLE..R..ASNM.L..L..I.I.TY...S.KQV.I......V.H.KEGKG | AAI37770.1 | | |
| g_aucja_scaff_4557_1.Contig17 | --K.I.....SA.LLE..R..STNM.L..I..I.ISKY...S.KQV.I......V.H.KEG-- | | 19 : 80662 | gsx |
